# A novel *LRAT* mutation affecting splicing in a family with early onset retinitis pigmentosa

**DOI:** 10.1186/s40246-018-0165-3

**Published:** 2018-07-04

**Authors:** Yabin Chen, Li Huang, Xiaodong Jiao, Sheikh Riazuddin, S. Amer Riazuddin, J. Fielding Hetmancik

**Affiliations:** 1 Ophthalmic Genetics and Visual Function Branch, National Eye Institute, National Institutes of Health, Bethesda, MD 20892 USA; 2 State Key Laboratory of Ophthalmology, Zhongshan Ophthalmic Center, Sun Yat-Sen University, Guangzhou, Guangdong China; 3 National Centre of Excellence in Molecular Biology, University of the Punjab, Lahore, Pakistan; 4 Allama Iqbal Medical College, University of Health Sciences, Lahore, Pakistan; 5 National Centre for Genetic Diseases, Shaheed Zulfiqar Ali Bhutto Medical University, Islamabad, Pakistan; 6 The Wilmer Eye Institute, Johns Hopkins University School of Medicine, Baltimore, MD USA

**Keywords:** Retinitis pigmentosa, *LRAT*, Splicing mutation, Cryptic splice site, Minigene assay, Exon splicing, Linkage

## Abstract

**Background and purpose:**

Retinitis pigmentosa is an important cause of severe visual dysfunction. This study reports a novel splicing mutation in the lecithin retinol acyltransferase (*LRAT*) gene associated with early onset retinitis pigmentosa and characterizes the effects of this mutation on mRNA splicing and structure.

**Methods:**

Genome-wide linkage analysis followed by dideoxy sequencing of the linked candidate gene *LRAT* was performed in a consanguineous Pakistani family with autosomal recessive retinitis pigmentosa. In silico prediction and minigene assays were used to investigate the effects of the presumptive splicing mutation.

**Results:**

ARRP in this family was linked to chromosome 4q31.21-q32.1 with a maximum LOD score of 5.40. A novel homozygous intronic mutation (NM_004744.4: c.541-15T>G) was detected in *LRAT*. In silico tools predicted that the AG-creating mutation would activate an intronic cryptic acceptor site, but cloning fragments of wild-type and mutant sequences of *LRAT* into Exontrap Cloning Vector pET01 and Expression Cloning Vector pCMV-(DYKD_4_K)-C showed that the primary effect of the sequence change was to weaken the nearby authentic acceptor site and cause exon skipping, with only a small fraction of transcripts utilizing the acceptor site producing the reference transcript.

**Conclusions:**

The c.541-15T>G mutation in *LRAT* results in aberrant splicing and is therefore predicted to be causal for the early onset retinitis pigmentosa in this family. In addition, this work suggests that minigenes adapted to the specific gene and exon may need to be designed for variants in the first and last exon and intron to mimic the authentic splicing mechanism in vivo.

**Electronic supplementary material:**

The online version of this article (10.1186/s40246-018-0165-3) contains supplementary material, which is available to authorized users.

## Introduction

Retinitis pigmentosa (RP, [MIM 268000]) is a clinically and genetically heterogeneous disorder affecting approximately 1 in 4000 individuals worldwide [1]. Clinically, patients initially exhibit night blindness followed by progressive loss of peripheral visual fields and eventually complete loss of central vision. Typical fundus changes include bone spicule-like pigmentation in the mid-peripheral retina, waxy pallor of the optic discs, and attenuation of retinal blood vessels. Since RP initially affects the rod photoreceptors, followed by the degeneration of cone photoreceptors, patients often have severely diminished or extinguished rod response in electroretinography (ERG) recordings even in early stages, while the cone response is relatively preserved initially but becomes undetectable as the disease progresses [2]. The genetic inheritance patterns of RP include autosomal-dominant (about 30–40% of cases), autosomal-recessive (50–60%), and X-linked (5–15%) [3, 4] inheritance. More than 82 causative genes have been identified for RP so far, of which 58 genes have been identified in families with autosomal recessive RP (arRP; RetNet).

Lecithin retinol acyltransferase (LRAT) is a retinyl ester synthase, catalyzing the formation of fatty acid retinyl esters, a crucial step in the retinoid cycle. In the eye, it is specifically expressed in the retinal pigmented epithelium (RPE), so that LRAT dysfunction leads to diminished visual chromophores and eventual retinal degeneration [5]. Mutations in the *LRAT* gene can cause Leber congenital amaurosis, juvenile retinitis pigmentosa, and early-onset severe retinitis pigmentosa with autosomal recessive inheritance (MIM 604863). Leber congenital amaurosis is the most severe retinal dystrophic disease. Patients usually present in the first decade of life with severe visual impairment and pendular nystagmus [6].

It is estimated that about 10% of disease-causing mutations affect splicing [7]. Most of the splice site mutations affect the invariant GT or AG dinucleotides in the 3′ and 5′ splice sites, respectively [8, 9]. However, mutations involving other positions of the 5′ or 3′ splice sites can also impair splicing and typically lead to exon skipping, activation of a cryptic splice site, or intron retention [10]. This study demonstrates that single base pair substitution in the intron even 15 bases upstream of 3′ splice site can disrupt spliceosomal recognition of the splice site.

## Materials and methods

### Clinical assessment

This study was approved by the Institutional Review Boards (IRB) of the National Centre of Excellence in Molecular Biology, Lahore, Pakistan, and the CNS IRB at the National Institutes of Health. Participating individuals or their guardians gave written informed consent consistent with the tenets of the Declaration of Helsinki before the study. Family 61254 is a consanguineous Pakistani family with non-syndromic RP. All participants underwent a thorough family, ophthalmic, and medical history, and selected individuals were assessed by best-corrected visual acuity, slit-lamp biomicroscopy, fundus photography, and electroretinography (ERG). Blood samples were collected from potentially informative family members, and genomic DNA was extracted from leukocytes according to standard protocols [11].

### Genome-wide linkage analysis

We designed linkage mapping panels to complete a genome-wide scan for family 61254. Primer sequences and PCR conditions are shown in Additional file 1: Table S1. Based on the initial results of the linkage analysis, markers with logarithm of the odds (LOD) scores greater than 2 were selected for further fine mapping by microsatellite markers closely spaced 1–2 cM apart.

Two-point linkage analyses were performed with alleles of family 61254 obtained through the genome-wide scan and fine mapping using the FASTLINK modification of the MLINK program in the LINKAGE program package [12, 13] using the pedegree file shown in Additional file 2. Maximum LOD scores were calculated using ILINK. Autosomal recessive RP was analyzed as a fully penetrant trait with an affected allele frequency of 0.0001. Haplotypes were drawn by the Cyrillic 2.1 program (Cyrillic Software, Wallingford, Oxfordshire, UK) and confirmed by inspection.

### Mutation screening

Primer pairs for the coding exons and 100 bp of flanking intronic regions of *LRAT* were designed using the Primer 3 program (http://primer3.ut.ee/) and are shown in Additional file 3: Table S3. Amplifications were completed in 10 μl reactions containing 40 ng of genomic DNA, 0.5 μl of 5 μM of each primer, 1 μl GeneAmp 10×PCR Gold buffer (Applied Biosystems), 0.6 μl of 25 mM MgCl2, 0.8 μl of 2.5 mM dNTP mixture, and 1 μl of 5 U/μl Taq DNA polymerase. PCR reactions consisted of a denaturation step at 95 °C for 5 min followed by a two-step touchdown procedure. The first 15 cycles consisted of denaturation at 95 °C for 30 s, followed by a touchdown annealing step for 30 s in which the annealing temperature was initially set at 64 °C and decreased by 0.5 °C per cycle, and an elongation step at 72 °C for 30 s. The second step of 20 cycles consisted of denaturation at 95 °C for 30 s followed by annealing at 57 °C for 30 s and elongation at 72 °C for 30 s. These were followed by a final elongation at 72 °C for 10 min. PCR products were purified using the AMPure XP system (Beckman coulter Biomek NX, Brea, CA). The PCR primers for each exon were used for bidirectional sequencing using the BigDye Terminator Ready Reaction mix (Applied Biosystems), according to the manufacturer’s instructions. The sequencing products were purified using the Agencourt CleanSEQ system (Beckman Coulter Biomeck NX). Sequencing was performed on an ABI PRISM 3130 Automated sequencer (Applied Biosystems, Foster City, CA). Sequencing results were analyzed using Mutation Surveyor (SoftGenetics, State College, PA) and Seqman software Lasergene 12.2.0 (DNASTAR, Madison, WI).

### In silico splice site prediction

To evaluate the potential pathogenicity of the splice site variation, several in silico splice site prediction programs were used, including Human Splice Finder (http://www.umd.be/HSF3/), SpliceView (http://bioinfo.itb.cnr.it/oriel/splice-view.html), Berkeley Drosophila Genome Project Neural Network (BDGP: http://www.fruitfly.org/), and NetGene2 (http://www.cbs.dtu.dk/services/NetGene2/). Default settings are applied for all analyses.

### Generation of vector constructs

Minigene assays were performed to confirm the predicted pathogenic effects of the *LRAT* mutation. Since mutation c.541-15T>G (NM_004744.4) was in intron 2, a 5228-bp genomic DNA fragment that included exon 2, intron 2, and part of exon 3 of *LRAT* was amplified using primers LRAT-gDNA-F and LRAT-gDNA-R. Because limited minigene amplicon length can be included in the minigene assay, and exon 3 is 4136-bp long with 153-bp coding sequence and 3983-bp 3′ UTR sequence, only part of exon 3 (402-bp) containing the coding region and several predicted downstream cryptic splice donor and acceptor sites were included in the 5228-bp fragment used for the minigene assay. The wildtype and mutant DNA fragments were amplified from genomic DNA of unaffected sibling 6125441 and patient 6125408 carrying the wild-type and homozygous mutant alleles, respectively. PCR products were cloned into a TA vector (pCR®II TOPO) and then subcloned into Exontrap cloning Vector pET01 (MoBiTec GmbH, Göttingen, Germany) via BamHI and NotI restriction sites to generate the wildtype WT-LRAT-pET01 and mutant Mut-LRAT-pET01 constructs.

Because exon 3 is the last exon of *LRAT*, to mimic authentic splicing mechanism in vivo, we also used a Q5 Site-Directed Mutagenesis Kit (New England Biolabs, Ipswich, MA) to abolish the splice acceptor site of 3′ exon on pET01 by altering the final acceptor AG to AT, creating the modified empty construct pET01-2, modified wildtype construct WT-2-LRAT-pET01, and modified mutant construct Mut-2-LRAT-pET01. In addition, the same 5228-bp genomic DNA fragment was also amplified by PCR with primers containing the appropriate restriction enzyme sites (LRAT-gDNA-F-SalI and LRAT-gDNA-R-KpnI) and then cloned into the expression vector pCMV-(DYKD_4_K)-C (Clontech Laboratories, Inc. CA, USA), which has no vector exons before and behind the inserted PCR products. SalI and KpnI restriction sites were used to insert the PCR products and construct wildtype construct WT-LRAT-pCMV and mutant construct Mut-LRAT-pCMV.

The primers used for PCR amplification and mutagenesis are listed in Table 1. The sequences and correct orientations of all constructs were validated using Sanger sequencing.

**Table 1:** Two-point LOD scores of markers around 4q31.21-q32.1

Marker	CM	Mb	0	0.01	0.05	0.1	0.2	0.3	0.4	*Z*_max_	*θ*_max_
D4S1615	128.31	128.21	− 3.81	− 2.00	− 0.36	0.15	0.30	0.17	0.03	0.30	0.20
D4S429	131.00	133.11	− 2.05	− 1.19	− 0.08	0.25	0.31	0.17	0.04	0.31	0.20
D4S1575	132.05	134.79	4.90	4.80	4.40	3.89	2.82	1.72	4.90	4.90	0.00
D4S3039	132.72	135.96	1.26	2.78	3.10	2.93	2.24	1.42	3.10	3.10	0.05
D4S1576	135.57	138.99	− 5.19	− 2.27	− 1.14	− 0.74	− 0.38	− 0.17	− 0.17	− 0.17	0.30
D4S1579	140.64	140.73	− ∞	− 0.01	1.01	1.17	0.89	0.42	1.17	1.17	0.10
D4S2939	142.24	141.15	− ∞	− 0.92	1.99	2.13	1.74	1.06	0.36	2.13	0.09
D4S424	144.56	142.20	− ∞	− 3.69	− 0.19	0.30	0.51	0.39	0.19	0.51	0.20
D4S2998	145.98	145.57	5.40	5.39	4.89	4.36	3.25	2.08	0.94	5.40	0.00
D4S1586	147.06	146.78	5.13	5.12	4.61	4.08	2.99	1.88	0.83	5.13	0.00
D4S3008	152.98	150.30	2.72	2.72	2.54	2.29	1.70	1.04	0.43	2.72	0.00
D4S1548	153.51	152.14	1.74	1.74	1.63	1.46	1.05	0.62	0.25	1.74	0.00
D4S3021	154.63	154.94	4.08	4.08	3.77	3.39	2.52	1.57	0.68	4.08	0.00
D4S3049	155.19	154.77	4.54	4.53	4.03	3.51	2.41	1.29	0.35	4.54	0.00
D4S2976	155.31	155.80	3.99	3.98	3.57	3.15	2.29	1.45	0.66	3.99	0.00
LRAT c.541-15T>G		155.67	6.57	6.47	6.04	5.48	4.28	2.94	1.49	6.57	0.00
D4S2934	155.41	154.05	4.89	4.88	4.40	3.90	2.86	1.81	0.83	4.89	0.00
D4S2918	157.99	160.33	2.77	2.76	2.45	2.14	1.51	0.91	0.39	2.77	0.00
D4S1585	157.99	157.51	3.05	3.04	2.74	2.42	1.78	1.13	0.52	3.05	0.00
D4S2982	158.65	161.09	− ∞	0.98	2.30	2.20	1.71	1.11	0.52	2.30	0.05

### Cell culture and transfection

Human embryonic kidney 293T cells and human retinal pigment epithelial cells (ARPE-19) were maintained in Dulbecco’s modified Eagle medium supplemented with 10% fetal bovine serum in 5% CO_2_ at 37 °C. The Exontrap cloning vectors pET01, WT-LRAT-pET01, Mut-LRAT-pET01, pET01-mut, WT-2-LRAT-pET01, and Mut-2-LRAT-pET01 were transfected into 293T cells using PolyJet In Vitro DNA Transfection Reagent (SignaGen Laboratories, MD). The expression vectors pCMV-(DYKD_4_K)-C, WT-LRAT-pCMV, and Mut-LRAT-pCMV were transfected into 293T cells and ARPE-19 cells, respectively, using PolyJet In Vitro DNA Transfection Reagent. For nonsense-mediated decay inhibition studies, non-transfected and transfected ARPE19 cells were treated with 0, 5 μM, and 10 μM PTC124 separately (Enzo Life Sciences, Inc., NY) for 24 h. Cells were harvested 24 h after transfection.

### RNA isolation, cDNA synthesis, PCR, and sequencing

Total cellular RNA was extracted using Trizol reagent (Life Technologies), and cDNA was synthesized using a reverse transcriptase kit (Invitrogen, Carlsbad, CA) with oligo (dT)_20_ primers. Primers used for PCR amplification for cDNA are listed in Additional file 2: Table S2. The amplified products were separated by electrophoresis on 10% Novex TBE gels (Invitrogen) and stained with ethidium bromide. Visualized bands were extracted from the gels, and the same primers were used for sequencing to validate the splice effects in the in vitro assays.

## Results

**Fig. 1:**
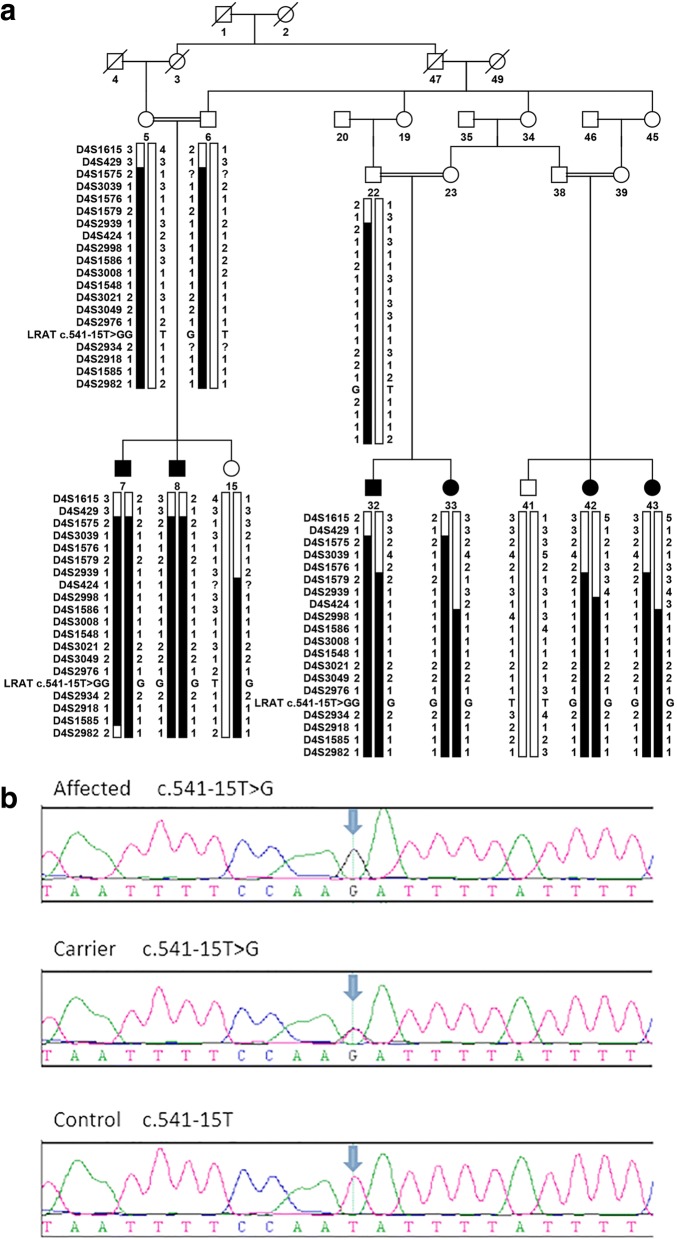
Pedigree, *LRAT* Region, and sequence. **a** Haplotypes of the *LRAT* region of family 61254 showing the *LRAT* c.541-15T>G mutation and surrounding microsatellite markers included in Table 1. The risk haplotype is marked in black. **b** Electropherograms of the *LRAT* homozygous mutation c.541-15T>G (up, individual 7), carrier sequence (middle, individual 22), and wildtype alleles (down, individual 41)

**Fig. 2:**
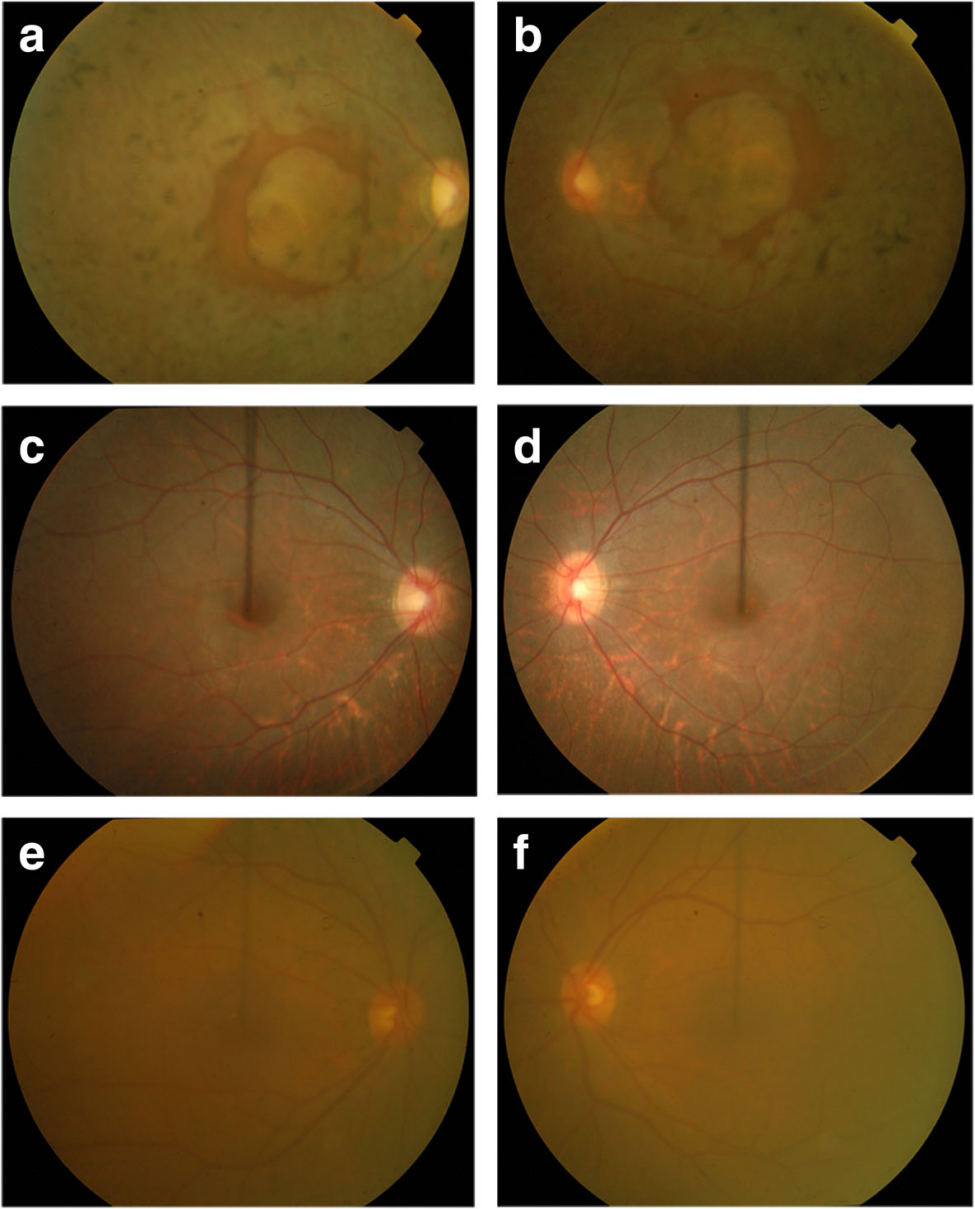
Fundus photographs of family 61254. **a**, **b** Oculus dexter (OD) and oculus sinister (OS) of an affected 35-year-old individual (08), which show waxy pale optic discs, attenuation of retinal arteries, bone spicule-like pigment deposits in the midperiphery of the retina, and macular changes. **c**, **d** OD and OS of an affected 10-year-old individual (32), which show attenuation of retinal arteries. **e**, **f** OD and OS of an unaffected 60-year-old individual (05), which show no signs of retinal dystrophy but does show haziness secondary to age-related cataracts

**Fig. 3:**
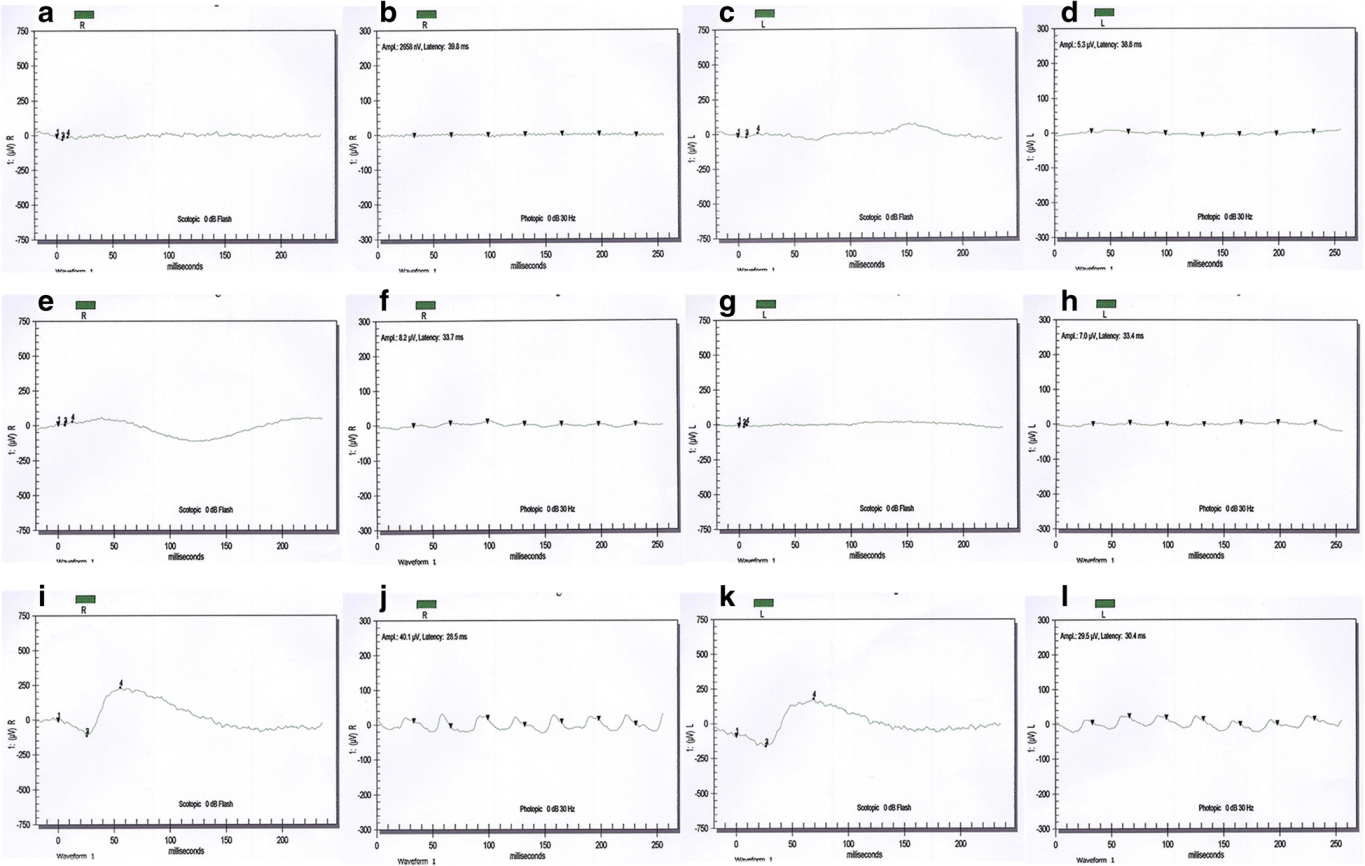
Electroretinography recordings of family members of 61254. **a**–**d** ERG responses of individual 8 (affected, 35-year-old). **e**–**h** ERG responses of individual 32 (affected, 10-year-old). **i**–**l** ERG response of individual 5 (unaffected, 60-year-old). **a**, **e**, and **i** OD combined rod and cone response; **b**, **f**, and **j** OD cone response; **c**, **g**, and **k** OS combined rod and cone response; **d**, **h**, and **l** OS cone response

RP in family 61254 showed an autosomal recessive inheritance pattern, and all affected individuals are offspring of first-cousin matings (Fig. 1a). The medical records of affected members in family 61254 reported signs of retinal degeneration early in life. Fundus photographs and ERGs were available only for individuals 5, 8, and 32 (Figs. 2 and 3). Affected individual 8, who was 35 years old at the time of examination, showed typical signs of RP, including waxy pale optic discs, attenuation of retinal arteries, bone spicule-like pigment deposits in the midperiphery of the retina, and macular changes. Affected individual 32, who was 10 years old at the time of examination, only showed attenuation of retinal arteries in fundus photographs. But both individual 8 and 32 had extinguished ERGs, consistent with extensive loss of rod and cone function typical of advanced arRP. Unaffected individual 5 showed no retinal abnormalities in fundus photographs and normal ERG recordings, although his fundus appears cloudy due to age-related cataracts.

During a genome-wide scan for family 61254, a LOD score greater than 2.0 was found only for markers D4S1575, D4S413, D14S261, and D17S921. Except for a single chromosome 4 locus, which was narrowed from D4S429 to D4S424, other regions were excluded by fine mapping markers at 1–2 cM intervals and haplotype inspection. Among the markers selected for fine mapping of the chromosome 4 locus, D4S2998 obtained a maximum LOD score of 5.40 at *θ* = 0 (Table 1). Thus, two-point linkage mapping in family 61254 identified a linked region of 14.09 cM (18.89 Mb) on chromosome 4q31.21-q32.1 flanked by markers D4S424 and D4S2982 (Table 1, Fig. 1a).

**Fig. 4:**
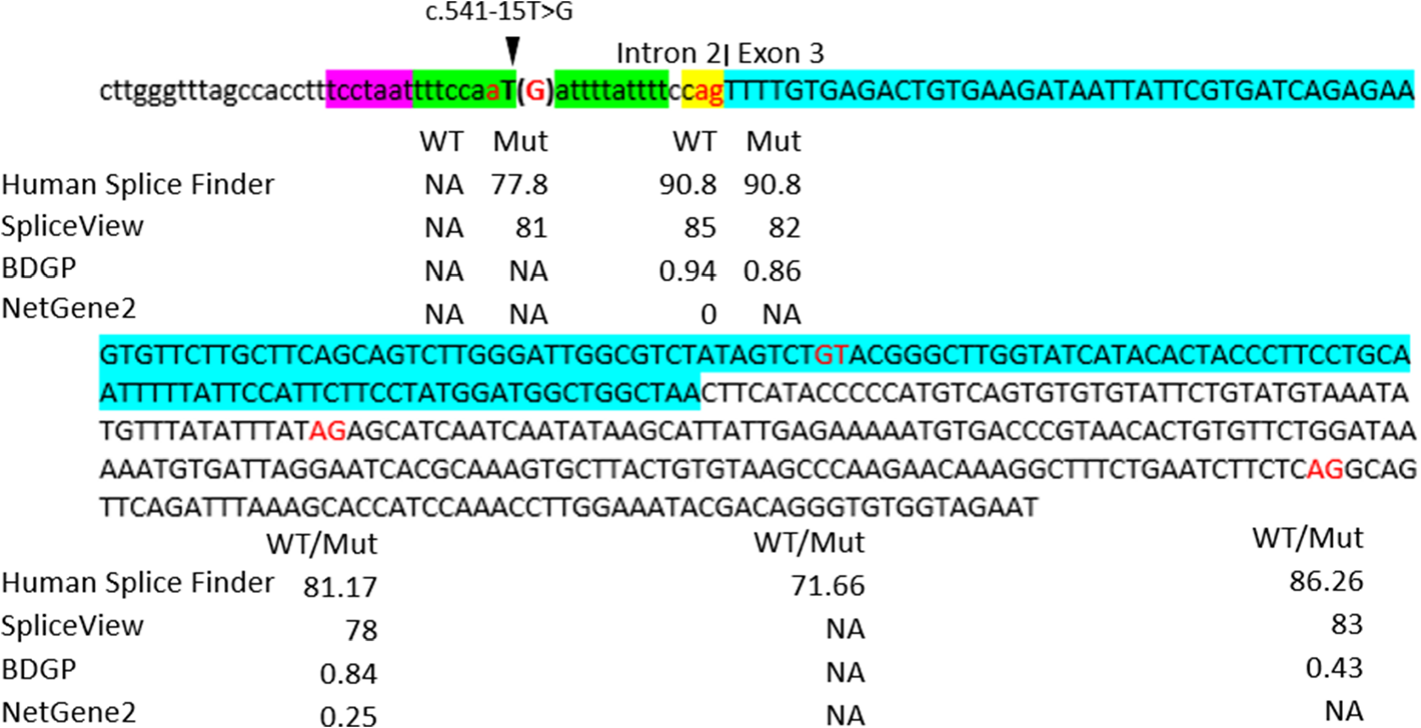
Results of in silico splice prediction tools. Lowercase nucleotides indicate intron 2 sequences, upper case nucleotides indicate exon 3 sequences, and the fragment highlighted in blue is the coding region. Nucleotides marked in red indicate potential splice donor or acceptor sites. The YAG sequence is shown in yellow, the polypyrimidine tract (PPT) is shown in green, and the branch point sequence (BPS) is shown in purple. The scores calculated for wildtype sequence (WT) and mutated sequence (Mut) using Human Splice Finder, SpliceView, BDGP, and NetGene2 are displayed below each splice site. A higher score implies greater potential for a splice site. NA means no splice site predictions above threshold of the in silico tool

*LRAT* is the only known candidate gene for inherited retinal diseases in the linked region. Sequencing of all coding exons and exon-intron boundaries detected a single novel splicing site variation in *LRAT*. All affected individuals in family 61254 carry a homozygous T>G change 5′ of exon 3 (c.541-15T>G, rs779487944), which cosegregates with the phenotype in the family (Fig. 1). The frequency of this change was 0.000004 as reported in the gnomAD database (http://gnomad.broadinstitute.org/about) with a single heterozygous individual in the South Asian population. In silico splice prediction tools including Human Splice Finder and SpliceView predicted that the AG-creating mutation would activate an intronic cryptic acceptor site, causing retention of an additional 14 bases from intron 2 in the transcript, leading to a frameshift in the translation reading frame, and resulting in a truncated protein p.(F181Ifs*10). The SpliceView program also predicted minimal weakening of the authentic acceptor site. However, the predicted new intronic cryptic acceptor site was not identified by BDGP or NetGene2, which only showed minimal weakening of the authentic acceptor site (Fig. 4).

**Fig. 5:**
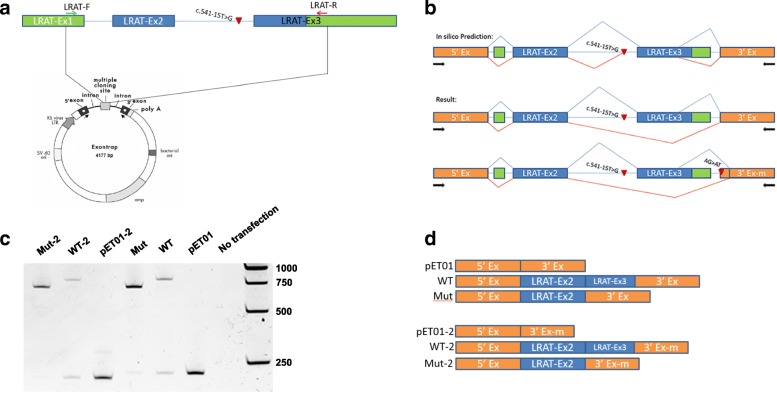
Splicing analysis using Exontrap pET01. **a** Diagram of *LRAT* genomic DNA, the location of the c.541-15T>G mutation and the fragment inserted into pET01 construct. In the pET01 construct, the multiple cloning site (MCS) is in an intron flanked by pre-proinsulin 5′ and 3′ exons. The primers used for cDNA amplification are within the pre-proinsulin 5′ and 3′ exons and indicated by black arrows. **b** Diagrams of transcript splicing of WT- and Mut- LRAT-pET01. Splicing events in the WT construct are shown by a blue line, and transcript splicing of c.541-15T>G Mut-LRAT-pET01 construct are shown by a red line. Results derived from in silico prediction (top), results obtained from minigene assay using pET01 vector (middle), and results obtained from minigene assay using pET01 vector with the splice acceptor site of 3′ exon abolished (bottom). **c** Electrophoresis results of cDNA amplification obtained from 293T cells transfected with empty construct pET01, wildtype construct (WT), c.541-15T>G mutant construct (Mut), modified empty construct (pET01-2), modified wildtype construct (WT-2), and modified c.541-15T>G mutant construct (Mut-2). The major PCR products of cDNA amplification from each construct as verified by sequencing are indicated in **d**

To test these predictions, we carried out a minigene assay with Exontrap Cloning Vector pET01 to examine the potential pathogenic effects of this mutation (Fig. 5a). The 293T cells transfected with empty plasmid (pET01) produced a 242-bp band, consistent with correct transcript splicing with excision of the intron flanked by pre-proinsulin 5′ and 3′ exons (Fig. 5c). Cells transfected with WT-LRAT-pET01 produced a faint 242-bp band and another 865-bp band. Direct sequencing of the PCR products revealed that the 865-bp band was consistent with correct mRNA splicing at the end 5′ of exon 3, which also utilized a cryptic donor site in the coding region of exon 3 of *LRAT* to join to the 3′ exon in pET01 (Fig. 5b, d). In contrast, cells transfected with Mut-LRAT-pET01 containing the c.541-15T>G mutation produced a shorter band that skipped exon 3 of LRAT. These findings are consistent with disruption of the authentic acceptor site of exon 3 by the c.541-15T>G change.

Because there is no exon following exon 3 in human genomic DNA of *LRAT*, we abolished the splice acceptor site of the pre-proinsulin 3′ exon on pET01 to reduce its effect on mRNA splicing of the inserted sequence of *LRAT*. Cells transfected with WT-2-LRAT-pET01 still produced mRNA with correct splicing at the 5′ end of exon 3, and cells transfected with Mut-2-LRAT-pET01 still produced mRNA skipping the exon 3. However, because the authentic acceptor site of the pre-proinsulin 3′ exon was abolished, the transcription utilized a cryptic acceptor site within the 3′ exon (Fig. 5).

**Fig. 6:**
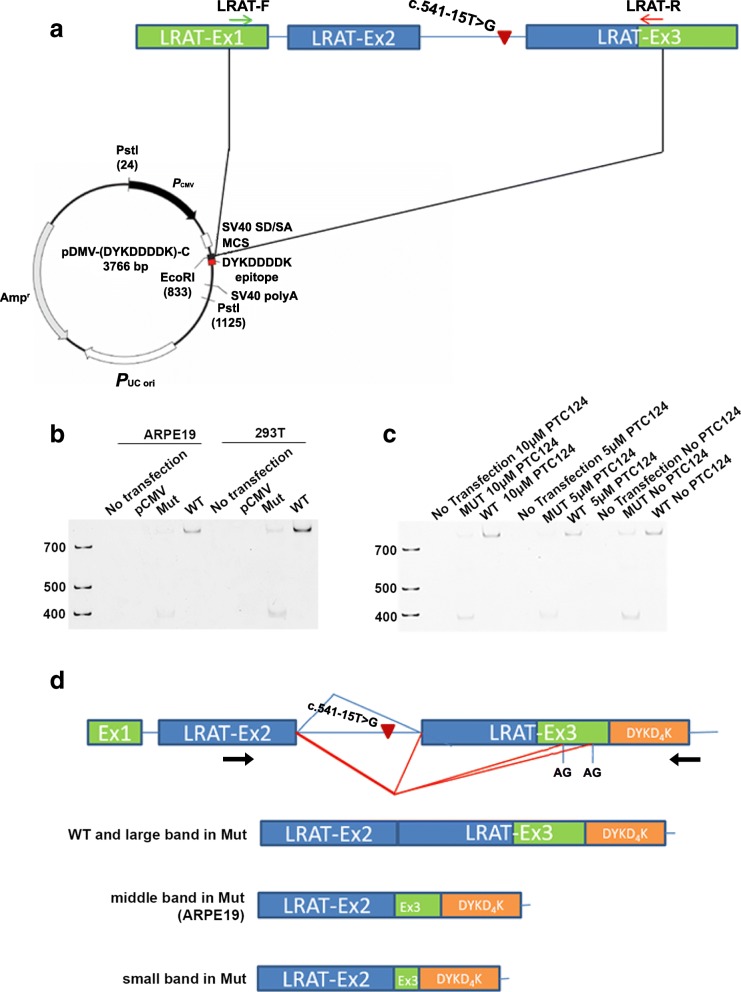
**a** Diagram of the *LRAT* gene showing the location of the c.541T>G variation and the fragment inserted into the pCMV-(DYKD_4_K)-C construct. **b** Electrophoresis results of cDNA amplification obtained from 293T cells and ARPE-19 cells transfected with the wildtype construct WT, mutated construct Mut, and empty construct pCMV. **c** Non-transfected and transfected ARPE19 cells were treated with 0, 5 μM, and 10 μM PTC124 separately for 24 h. There were no extra transcripts under PTC124 treatment. **d** Diagram of transcript in which splicing of WT-LRAT-pCMV is shown by a blue line; transcript splicing of Mut-LRAT-pCMV is shown by a red line. The forward primer used in cDNA amplification is within the exon 2 of *LRAT* and the reverse primer binds to the construct sequence of DYKD_4_K epitope, which are indicated by arrows (the AG shown in the 3′ UTR of LRAT refers to the last two AG in Fig. 4). A diagram of the PCR product of cDNA amplification from each construct is also shown in **d**

To further mimic the splicing mechanism in vivo, we inserted the same 5228-bp genomic DNA fragment of *LRAT* into expression vector pCMV-(DYKD_4_K)-C which has no exons before or behind the inserted region and used it to transfect both 293T cells and ARPE19 cells (Fig. 6a). Cells transfected with WT-LRAT-pCMV produced a 723 bp cDNA with correct splicing at the 5′ end of exon 3 (Fig. 6b, d). Both 293T cells and ARPE19 cells transfected with Mut-LRAT-pCMV produced a much fainter band of the same size, as well as a shorter 379 bp fragment skipping the authentic splice acceptor site at the 5′ of exon 3 and utilizing a cryptic acceptor site in the 3′ UTR instead. In contrast to the results in 293T cells, ARPE19 cells transfected with Mut-LRAT-pCMV also produced an extremely faint 513 bp fragment skipping the authentic splice site at the 5′ of exon 3 and utilizing another cryptic acceptor site in the 3′ UTR. To investigate whether the mutation caused mis-splicing events which would lead to nonsense-mediated decay, non-transfected and transfected ARPE19 cells were treated with PTC124 for 24 h to inhibit nonsense-mediated decay, and no extra transcripts were identified (Fig. 6c).

## Discussion

We have identified a c.541-15T>G mutation in *LRAT* that results in aberrant splicing as the cause of autosomal recessive early-onset retinitis pigmentosa in a Pakistani family. The mammalian 3′ splice site consensus consists of three critical parts: YAG (Y = pyrimidine) at positions − 1 to − 3 relative to the first nucleotide of exon, a polypyrimidine tract (PPT) starting at position − 5 and extending 10 or more nucleotides into the intron, and the branch point sequence (BPS; mammalian consensus YNCURAY, where Y = pyrimidine, R = purine, and N = any nucleotide) located upstream of the PPT, usually 11–40 nucleotides from the YAG [14, 15]. The PPT is a crucial recognition element for branch site definition prior to splicing, and the factor responsible for this early PPT recognition is U2AF,^65^ a subunit of the U2 snRNP auxiliary factor (U2AF) [14]. Since the c.541-15T>G mutation replaces a pyrimidine with a purine at the 5′ end of the PPT, it might possibly affect the interaction of the U2AF^65^ with the PPT, thus weakening activity of the authentic splice acceptor site. Both bioinformatic tools and minigene assays showed that the novel c.541-15T>G mutation in the *LRAT* gene causes aberrant splicing and is therefore causal for early onset retinitis pigmentosa, further broadening the mutation spectrum in the *LRAT* gene.

Two in silico prediction tools, Human Splice Finder and SpliceView, predicted creation of a new splice acceptor site by the mutation, consistent with the AG/GT rule. Minigene assays using both vector pET01 and pCMV-(DYKD_4_K)-C based constructs showed that the resulting mutant cDNA skipped exon 3 of *LRAT*. The wild-type transcript was absent in 293T cells transfected with the pET01 construct containing the mutant fragment of *LRAT*, and identified at reduced levels in 293T cells transfected with mutated pCMV-(DYKD_4_K)-C construct compared with cells transfected with the wildtype pCMV construct. This suggests that the mutation does not completely inactivate the authentic splice site but rather suppresses it so that some WT *LRAT* RNA might be present, consistent with the concept of leaky splicing [16]. This RNA level might not be sufficient to maintain adequate *LRAT* function in the retina so that patients homozygous for this mutation might manifest a RP phenotype. Although the minigene assays are not in perfect accordance with the in silico predictions about splicing patterns, all indicated that the mutation would result in aberrant splicing.

Minigene assays provide a rapid way to evaluate the impact of splice site mutations, especially when isolation and characterization of mRNA from the original source is technically difficult or ethically impossible. *LRAT* is expressed in RPE cells and is important for the retinoid cycle [5]. Since it is not expressed in peripheral blood cells and retinal biopsy is too invasive for patients, minigene assays provided an optimal approach to studying the in vitro effect of *LRAT* spice site mutations. However, the system also does not always reflect the exact splicing patterns of in vivo studies. While it allows prediction of whether a mutation is going to influence splicing, several studies have found occasional differences in splice patterns between minigene and RNA analyses in patient samples [17–19]. This might well relate to differences between the vector sequence surrounding the cloned insert and the genomic DNA in vivo. In an attempt to address this, we abolished the splice acceptor site of 3′ exon on pET01 and also used the pCMV-(DYKD_4_K)-C vector to investigate further the splicing effect of the mutation, aiming to weaken the influence of vector sequence on the cloned insert. The results confirmed that the mutation could cause splicing impairment compared with the wildtype sequence.

In conclusion, bioinformatics tools may be useful for predicting the pathogenicity of variants, but minigene assays are more reliable in the evaluation of the potential effects of splice-site mutations. Adapted minigenes may need to be designed for variants in the first and last exon and introns to mimic the authentic splicing mechanism in vivo. Despite the limitations of both in silico predictions and minigene assays, it is likely that this mutation c.541-15T>G is pathogenic, when all the information including linkage and co-segregation in the pedigree excluding other candidates, the known role of LRAT in retinal degeneration, in silico analyses predicting splicing impairment, and two minigene assays are consistent with weakening of the authentic splice acceptor site resulting in a truncated LRAT protein and arRP.

## Additional files

Additional file 1: **Table S1.** Primer sequences and PCR conditions of linkage mapping panels, A. Marker specific primers, B. Primers with fluorescent tags, C. Amplification Protocol (DOCX 32 kb).

Additional file 2: **Table S2.** Primer list (DOCX 12 kb).

Additional file 3: **Table S3.** LINKAGE profile of Family 61,254 (XLSX 13 kb).
