# Supplementary material for: A novel LRAT mutation affecting splicing in a family with early onset retinitis pigmentosa
Source: Hum Genomics. 2018 Jul 4;12:35. doi: 10.1186/s40246-018-0165-3 (PMC6033202; doi:10.1186/s40246-018-0165-3)
Supplement: Supplementary file 1 — Table S1. Primer sequences and PCR conditions of linkage mapping panels, A. Marker specific primers, B. Primers with fluorescent tags, C. Amplification Protocol (DOCX 32 kb). [file 40246_2018_165_MOESM1_ESM.docx]

**Table S1. Primer sequences and PCR conditions of linkage mapping panels**

**A. Marker specific primers**

| **Panel** | **Markers** | **Forward primer (5'>3')** | **Reverse primer (5'>3')** | **Protocol*** | **Tag** |
| --- | --- | --- | --- | --- | --- |
| 1 | D1S2797 | GACAGCATCGCCAGTCACTAATCACATCACACACAATGACTGTGG | GCGTAGAGGATCCACAGGACTGTCCATTCAAAGGATTGGTCTC | TD | P1 |
| 1 | D1S450 | GACAGCATCGCCAGTCACTACACCTTCCTCTGCCTTTTTG | GCGTAGAGGATCCACAGGACAGCCCCCATGATGGTTAAAT | TD | P1 |
| 1 | D1S249 | GACAGCATCGCCAGTCACTAGAGGGAAATCCCATCTCAAA | GCGTAGAGGATCCACAGGACTCAAAATGTGTTACCAAATGTGC | TD | P1 |
| 1 | D1S446 | GACAGCATCGCCAGTCACTAGGCAGAGGTGGAAATACAGC | GCGTAGAGGATCCACAGGACGCCAATTTCCAAGAATCCAA | TD | P1 |
| 1 | D1S234 | GACAGCATCGCCAGTCACTAGCCTGTAGTCCCAGCTACTCA | GCGTAGAGGATCCACAGGACTTTCCGCAACCACTATTTAGC | TD | P1 |
| 1 | D1S255 | GCTTCCTAATGCAGGAGTCGTGTCAACCCACAAATCCAGA | GTGCAAGATTCCGAATACCGTTACGTGTTTTGGTGATGGTG | TD | P2 |
| 1 | D1S2785 | GCTTCCTAATGCAGGAGTCGGGGATGGTCTCAGTCTCCTG | GTGCAAGATTCCGAATACCGCCTGGCCAAAATGATGAAAC | 59 | P2 |
| 1 | D1S484 | GCTTCCTAATGCAGGAGTCGTCTGAAATTAAATCATTGCTCCA | GTGCAAGATTCCGAATACCGTTCCAAAGAATGCACGTAACA | TD | P2 |
| 1 | D1S2890 | GCTTCCTAATGCAGGAGTCGCGAATGGCCATCCTGTCTAT | GTGCAAGATTCCGAATACCGTGTGCAAGATGTTTGCTGTG | TD | P2 |
| 1 | D1S2667 | GCTTCCTAATGCAGGAGTCGCAGGACCCTGGAAGATGAAC | GTGCAAGATTCCGAATACCGATTCGGTGTGGAGCTCAAGT | TD | P2 |
| 1 | D1S213 | TTCAACCCAGTCAGCTCCTTCATTATCCAAGGTCAGGAGG | ACCAGTGACGAAGGCTTGAGAGCTGTTAATCCAATCTATGATGTG | TD | P3 |
| 1 | D1S2726 | TTCAACCCAGTCAGCTCCTTTCTGCCAGTCATGAAATGCT | ACCAGTGACGAAGGCTTGAGTGAAAGATCGTGTGTGACTAAACA | 59 | P3 |
| 1 | D1S2878 | TTCAACCCAGTCAGCTCCTTATTGGCTGAGGATGAGGTTG | ACCAGTGACGAAGGCTTGAGTTGCATAAGAAAGCAATGTTCC | 59 | P3 |
| 1 | D1S2836 | TTCAACCCAGTCAGCTCCTTGGGGACGTGGAGGTTAAAGT | ACCAGTGACGAAGGCTTGAGACCTTTGCAAGCCTCTGCT | 59 | P3 |
| 1 | D1S2842 | TTCAACCCAGTCAGCTCCTTTCACCTGACCTGTCCCTTAAA | ACCAGTGACGAAGGCTTGAGATACCATCCTCCGCAGAAGC | TD | P3 |
| 1 | D1S206 | TTCAACCCAGTCAGCTCCTTCACCTTCTCGTCAGGCAAAT | ACCAGTGACGAAGGCTTGAGTGGAGAATCTGTCTCCAAACTG | 59 | P3 |
| 2 | D1S199 | ATCAGGGACAGCTTCAAGGAGGTGACAGAGTGAGACCCTG | GGAGTGGTGAATCCGTTAGCCAAAGACCATGTGCTCCGTA | TD | P4 |
| 2 | D1S207 | ATCAGGGACAGCTTCAAGGACACTTCTCCTTGAATCGCTT | GGAGTGGTGAATCCGTTAGCGCAAGTCCTGTTCCAAGTCT | TD | P4 |
| 2 | D1S468 | ATCAGGGACAGCTTCAAGGAACCAGTACATGCTTCCAGCAG | GGAGTGGTGAATCCGTTAGCGCTCCTCAAAATCCTCTGACC | TD | P4 |
| 2 | D1S413 | ATCAGGGACAGCTTCAAGGAGCCAAGCCTGAGATCAAAAT | GGAGTGGTGAATCCGTTAGCACTTGAACAGATTGGGATTG | TD | P4 |
| 2 | D1S238 | ATCAGGGACAGCTTCAAGGAATGAATCAAATTCAGCCATGC | GGAGTGGTGAATCCGTTAGCACAGATGGTGCAAAATGAAGC | TD | P4 |
| 2 | D1S252 | GGCATTGACCCTGAGTGATTAGCTTTTTACTCTTAACCTATTCAT | ACTGATGCCTCCGTGTAAGGGCAGAGAACATGTGTATTAATGA | 59 | P5 |
| 2 | D1S2868 | GGCATTGACCCTGAGTGATTAGGTATAATCTGCAATAAAAAACTT | ACTGATGCCTCCGTGTAAGGAAAGTAAAACAATATGAAGCCAC | TD | P5 |
| 2 | D1S230 | GGCATTGACCCTGAGTGATTCCTGGCAGATCAAGGCT | ACTGATGCCTCCGTGTAAGGAGCTGCATGTAAGTGCTGAG | TD | P5 |
| 2 | D1S2697 | GGCATTGACCCTGAGTGATTAAGGCTGTAGGGAGCTGTGAT | ACTGATGCCTCCGTGTAAGGAGTTTTCCATTTGACCTCACG | TD | P5 |
| 2 | D1S2841 | GGCATTGACCCTGAGTGATTAGCAAGCAAGCAAGTGAAAAC | ACTGATGCCTCCGTGTAAGGGGGACAGAAGGAGACCCTCTA | TD | P5 |
| 2 | D1S425 | ACGTAGCGATAGCGGAGTGTCTAAAACAAAACACTCAGAACCACA | GAGTCAGTGAGCGAGGAAGCACCTAGCACAAGGCTTCACA | TD | P6 |
| 2 | D1S2870 | ACGTAGCGATAGCGGAGTGTGACCCTGCCTCAAAACAAAA | GAGTCAGTGAGCGAGGAAGCTCCTCCTACCTTGGCCTTCT | TD | P6 |
| 2 | D1S498 | ACGTAGCGATAGCGGAGTGTTTGCTGAAGGGACATAGTG | GAGTCAGTGAGCGAGGAAGCTGCTGGGTTATATCCAATATC | TD | P6 |
| 2 | D1S218 | ACGTAGCGATAGCGGAGTGTTGTAAAAGCAAACTGTAGACGAT | GAGTCAGTGAGCGAGGAAGCTTTATGTTATCACCAAGGCTTCT | TD | P6 |
| 3 | D2S286 | CGGCATCAGAGCAGATTGTATAAAATTGTTTCTATGACATGATG | AACCGTATTACCGCCTTTGAATGGTGGTTTATCTTACCAGTC | 59 | P7 |
| 3 | D2S160 | CGGCATCAGAGCAGATTGTATGTACCTAAGCCCACCCTTTAGAGC | AACCGTATTACCGCCTTTGATGGCCTCCAGAAACCTCCAA | TD | P7 |
| 3 | D2S2211 | CGGCATCAGAGCAGATTGTATGGGTTAGTCATCAAGGGA | AACCGTATTACCGCCTTTGAGATTCAGGAGTCTGGGAAGT | TD | P7 |
| 3 | D2S367 | CGGCATCAGAGCAGATTGTATCCTCTCCACTGCACCTCTT | AACCGTATTACCGCCTTTGACTCAGCACATGTGTCTCCCTA | TD | P7 |
| 3 | D2S165 | CGGCATCAGAGCAGATTGTAAACTTCTACCTTGGCTGTGG | AACCGTATTACCGCCTTTGATTCAACTCCTTTGAGATTGT | 59 | P7 |
| 3 | D2S125 | GGTGATGACGGTGAAAACCTGCAACAGAGTGAGACCCTGA | CGCATATGGTGCACTCTCAGTTCTGAGAACCAGATTGTGATTG | TD | P8 |
| 3 | D2S206 | GGTGATGACGGTGAAAACCTTTAAAAATTAAGTAGGCTTTTGGTT | CGCATATGGTGCACTCTCAGGTCCTCATGTGTTTATGCTGT | TD | P8 |
| 3 | D2S117 | GGTGATGACGGTGAAAACCTGAGATCAGGTATATTCAATCCAC | CGCATATGGTGCACTCTCAGCAGAAAATGACAAACTTTAGAGAG | TD | P8 |
| 3 | D2S142 | GGTGATGACGGTGAAAACCTTGGAGACACTGAACGGGTAG | CGCATATGGTGCACTCTCAGTGAAAAGTTTTGAGATGGAGAGATT | TD | P8 |
| 3 | D2S325 | GGTGATGACGGTGAAAACCTCACATCTTTATTCAAGGGTTCAA | CGCATATGGTGCACTCTCAGTTCACTGTTGGCTGCATTTC | TD | P8 |
| 3 | D2S126 | GGCGCTTTCTCATAGCTCACTGCCTAGCACAGAGCCTACT | GCCTACATACCTCGCTCTGCTCCATGGCATAGAACAAAAGT | 59 | P9 |
| 3 | D2S337 | GGCGCTTTCTCATAGCTCACTCTGCATTCCCACGATTACA | GCCTACATACCTCGCTCTGCTTTCCCCTGTCCTTTCCAC | TD | P9 |
| 3 | D2S2333 | GGCGCTTTCTCATAGCTCACAGAAACCATGCCCTTG | GCCTACATACCTCGCTCTGCTGCAGTCACCTGTGTAGAA | 59 | P9 |
| 3 | D2S364 | GGCGCTTTCTCATAGCTCACCCCCTCTTGGGAAAACATT | GCCTACATACCTCGCTCTGCTCCCTCCAGAGCAAAAAGTG | 59 | P9 |
| 4 | D2S112 | ATCAGGGACAGCTTCAAGGAAACCCAGTCCCTGAGTGATTC | GGAGTGGTGAATCCGTTAGCGTGTGTCCAGCTGAGAGTTCC | TD | P1 |
| 4 | D2S2330 | GACAGCATCGCCAGTCACTATTGTCCATGTGCTATACTGCAA | GCGTAGAGGATCCACAGGACATATGGGTTTGAATCATAGTTTTACA | TD | P1 |
| 4 | D2S2259 | GACAGCATCGCCAGTCACTACAGCCTGTTTGGGGACAC | GCGTAGAGGATCCACAGGACGCAGAGCCATCGCCTTC | TD | P1 |
| 4 | D2S162 | GACAGCATCGCCAGTCACTACCAATTCAGAGATGGGTTTGA | GCGTAGAGGATCCACAGGACCCTCACTGGTAGACAGGAACG | TD | P1 |
| 4 | D2S2216 | GACAGCATCGCCAGTCACTAGGAACATGCGTAGCTCCATC | GCGTAGAGGATCCACAGGACCAGTTCCTGGGAACAAGCAT | TD | P1 |
| 4 | D2S347 | GACAGCATCGCCAGTCACTAACTGCATGAGCCAATTCCTT | GCGTAGAGGATCCACAGGACCATTTGCCCAATAAGGAGCTA | 59 | P1 |
| 4 | D2S319 | GCTTCCTAATGCAGGAGTCGGTAGACGTGGTGACACAGTTACAA | GTGCAAGATTCCGAATACCGCTAAATCGCATGCATTGCCT | TD | P2 |
| 4 | D2S168 | GCTTCCTAATGCAGGAGTCGCCGGACAACAGAGCGA | GTGCAAGATTCCGAATACCGAGCTCATTGCTATGGGAGTA | TD | P2 |
| 4 | D2S2382 | GCTTCCTAATGCAGGAGTCGGGTCCTCTCAGAAGTCAAACG | GTGCAAGATTCCGAATACCGCTTTTGTTGCCATCCAGTGA | TD | P2 |
| 4 | D2S151 | GCTTCCTAATGCAGGAGTCGTGCTATTAAAACAGACAGATGAGGA | GTGCAAGATTCCGAATACCGCCCACCTGAAACCAGAACTT | TD | P2 |
| 4 | D2S362 | TTCAACCCAGTCAGCTCCTTAAAAACAACTCTGAATGTAGTCTGAAA | ACCAGTGACGAAGGCTTGAGCGGTTCCTAACAGCATTACCA | 59 | P3 |
| 4 | D2S391 | TTCAACCCAGTCAGCTCCTTATGGAGCCAGTAGGTTACAGC | ACCAGTGACGAAGGCTTGAGGGTGAGAGGGTATGATGGAA | 59 | P3 |
| 4 | D2S335 | TTCAACCCAGTCAGCTCCTTTGAGAGCACAGACTCACTTGG | ACCAGTGACGAAGGCTTGAGTCACTGCCAATATCACCATCA | 59 | P3 |
| 4 | D2S338 | TTCAACCCAGTCAGCTCCTTCAAAACCCCGCCTCTACTAA | ACCAGTGACGAAGGCTTGAGGCATTTCTAATGGAAGTCATGC | TD | P3 |
| 4 | D2S305 | TTCAACCCAGTCAGCTCCTTGGCCCACACTGAAGACTTTG | ACCAGTGACGAAGGCTTGAGACAGGCATGGCAGACACC | TD | P3 |
| 5 | D4S392 | ATCAGGGACAGCTTCAAGGATCGGTAAACATTCATCCAGA | GGAGTGGTGAATCCGTTAGCTGTCAAAATGGACCAATCAG | TD | P4 |
| 5 | D3S1311 | ATCAGGGACAGCTTCAAGGAGGAAGTTTCAGCCAACG | GGAGTGGTGAATCCGTTAGCTTAGTCCCACTGATGTTACATTT | TD | P4 |
| 5 | D4S406 | ATCAGGGACAGCTTCAAGGATCACAGATATTCTAAATCCTCCAGTG | GGAGTGGTGAATCCGTTAGCTGTGACTTAAGCCATCCCATC | TD | P4 |
| 5 | D3S1565 | ATCAGGGACAGCTTCAAGGATATCTCTGATTAAAAAACCTCCTCC | GGAGTGGTGAATCCGTTAGCTGCAAGGGTGCCATCT | TD | P4 |
| 5 | D4S1575 | ATCAGGGACAGCTTCAAGGAGGAAGTTGAGGCATAAAAATCTC | GGAGTGGTGAATCCGTTAGCTTTATGGGTACTTTTTGAATCACAT | TD | P4 |
| 5 | D3S1271 | GGCATTGACCCTGAGTGATTTGATTGGAGGTGGTAGAGGT | ACTGATGCCTCCGTGTAAGGAGCTATCATGTAGAAAAGCAGCA | TD | P5 |
| 5 | D3S3681 | GGCATTGACCCTGAGTGATTTTGTGTTTTCAAACATCGCTAAA | ACTGATGCCTCCGTGTAAGGGAAGCATGAATTCTTCTTTTCAGTC | TD | P5 |
| 5 | D4S414 | GGCATTGACCCTGAGTGATTTTGCACAAAGCATCAGCC | ACTGATGCCTCCGTGTAAGGTCAGGAACCTCAGCCCAT | TD | P5 |
| 5 | D4S405 | GGCATTGACCCTGAGTGATTATGTTGCCTTGCCCTTTTACT | ACTGATGCCTCCGTGTAAGGCCCATGATCTTTCCTCAAACA | TD | P5 |
| 5 | D4S1534 | ACGTAGCGATAGCGGAGTGTGCAGGTTACTCCCAGGACAGT | GAGTCAGTGAGCGAGGAAGCTAGACCAGCCCAAGGTAGAGG | TD | P6 |
| 5 | D3S1614 | ACGTAGCGATAGCGGAGTGTCCAATCTTCATTTCCTCTTCCA | GAGTCAGTGAGCGAGGAAGCTAAGCCTTGACCCTGAACTTG | TD | P6 |
| 5 | D3S1285 | ACGTAGCGATAGCGGAGTGTACACAGCTCACGAAAAGGTAAAT | GAGTCAGTGAGCGAGGAAGCCCCTAGAACTTCCCCAGAAGTAG | TD | P6 |
| 5 | D3S1263 | ACGTAGCGATAGCGGAGTGTCTGTTGACCCATTGATACCC | GAGTCAGTGAGCGAGGAAGCTAAAATCACAGCAGGGGTTC | 59 | P6 |
| 5 | D4S1597 | ACGTAGCGATAGCGGAGTGTTATAGTGGCCCCTAGTGTTACAT | GAGTCAGTGAGCGAGGAAGCTTAGGCTTCTCAAAGCATAAGAC | TD | P6 |
| 6 | D3S1262 | CGGCATCAGAGCAGATTGTAAAAGAATGCTGGATAGCCAAA | AACCGTATTACCGCCTTTGAACGAATTATTTGAGCTCCAGTTT | 59 | P7 |
| 6 | D4S413 | CGGCATCAGAGCAGATTGTATCTGAATATAGTGCTCCAGAAA | AACCGTATTACCGCCTTTGACAATCAGTGGGTTTTTGAA | TD | P7 |
| 6 | D4S1572 | CGGCATCAGAGCAGATTGTACACCATGCCAAGCCTTCTTA | AACCGTATTACCGCCTTTGACACCACTGCACTCCATTCTG | TD | P7 |
| 6 | D3S1300 | CGGCATCAGAGCAGATTGTAAGCTCACATTCTAGTCAGCCT | AACCGTATTACCGCCTTTGAGCCAATTCCCCAGATG | TD | P7 |
| 6 | D3S1569 | CGGCATCAGAGCAGATTGTATTGCCATAATTTCCCAAACA | AACCGTATTACCGCCTTTGAAAATGCTGGGATCCAGAAAG | TD | P7 |
| 6 | D4S2935 | GGTGATGACGGTGAAAACCTTGCCCAATAAATGGCAGTTC | CGCATATGGTGCACTCTCAGCAGCCCTGGGTGAAGTTTA | TD | P8 |
| 6 | D4S391 | GGTGATGACGGTGAAAACCTATATCCCAGTTACCCCAATTTGA | CGCATATGGTGCACTCTCAGGCAGTCACATGTTAGGTCAGAG | TD | P8 |
| 6 | D4S1592 | GGTGATGACGGTGAAAACCTGATTGTACCACTGCCCTCC | CGCATATGGTGCACTCTCAGCCACCATACCTGGCCTTG | 59 | P8 |
| 6 | D3S1297 | GGTGATGACGGTGAAAACCTTGCACATTAAAGGAACAGGT | CGCATATGGTGCACTCTCAGCATAATTTGCTGCTTTGGAT | TD | P8 |
| 6 | D3S1304 | GGTGATGACGGTGAAAACCTTTCGCTCTTTGATAGGC | CGCATATGGTGCACTCTCAGATTTCATTTGTAATTTACTAGCAG | TD | P8 |
| 6 | D3S1601 | GGTGATGACGGTGAAAACCTTGCAGGCACCTTATAACAGGA | CGCATATGGTGCACTCTCAGAGCCAGGTGGTTCTGTGAAT | TD | P8 |
| 6 | D3S1292 | GGCGCTTTCTCATAGCTCACGAGGTAAGCTGTGGACCCTG | GCCTACATACCTCGCTCTGCGTGATTCCAAATAACCTGCAACA | 59 | P9 |
| 6 | D4S426 | GGCGCTTTCTCATAGCTCACATACACTGCATCCATATATACAAGG | GCCTACATACCTCGCTCTGCACATTGTGAAATGACCACAG | TD | P9 |
| 6 | D4S419 | GGCGCTTTCTCATAGCTCACCCATCCCTCCACAGTTCTCT | GCCTACATACCTCGCTCTGCGTTGGATTTTGAAGCAGTGTTTA | TD | P9 |
| 6 | D4S415 | GGCGCTTTCTCATAGCTCACGGGCAAGATGGGTTGAAGT | GCCTACATACCTCGCTCTGCTTTTGAAAGACATTTCCCTAAGGT | TD | P9 |
| 7 | D3S2338 | GACAGCATCGCCAGTCACTACTGTGGCCATCTTCTCCTGT | GCGTAGAGGATCCACAGGACGCTGAATTTTCTGTATTGTTTTCC | 59 | P1 |
| 7 | D3S1580 | GACAGCATCGCCAGTCACTAGACAGCTTTCTTCACCACCA | GCGTAGAGGATCCACAGGACTGGTAAAGTCATAGAATAGTGACCA | 59 | P1 |
| 7 | D4S403 | GACAGCATCGCCAGTCACTAAGGTGGCCCTGAGTAGGAGT | GCGTAGAGGATCCACAGGACTTTGAGGGAATGATTTGGGT | 59 | P1 |
| 7 | D4S402 | GACAGCATCGCCAGTCACTACTTACTGTGTTGCCCAAGGT | GCGTAGAGGATCCACAGGACAGCTCTATGATTCATTTCAAGTTTG | TD | P1 |
| 7 | D4S1539 | GACAGCATCGCCAGTCACTAGGTCGTCTCTGCGATAGAGC | GCGTAGAGGATCCACAGGACGGACTTACAGCATCAGGCATT | 59 | P1 |
| 7 | D4S2964 | GCTTCCTAATGCAGGAGTCGTTCCCAACTCACACATGCAC | GTGCAAGATTCCGAATACCGTGGTATAGCATGGGCACAAA | 59 | P2 |
| 7 | D4S412 | GCTTCCTAATGCAGGAGTCGGGCTTCATCCATGTTGTTGC | GTGCAAGATTCCGAATACCGAGGGATAAAGGAAATGTGCGA | 59 | P2 |
| 7 | D3S1266 | GCTTCCTAATGCAGGAGTCGCCCTAAGGAGGGCTAGAACC | GTGCAAGATTCCGAATACCGAGTTGTCATGGAGATGGCATT | 59 | P2 |
| 7 | D3S1278 | GCTTCCTAATGCAGGAGTCGATTTGCCTGAGGGAGGAGAT | GTGCAAGATTCCGAATACCGCCACTGCTTCCTGTCTCCAT | 59 | P2 |
| 7 | D4S424 | GCTTCCTAATGCAGGAGTCGCAGCACTTCCCTTTATCTTTGC | GTGCAAGATTCCGAATACCGTGCAGCCTAGCAATCTTGG | 59 | P2 |
| 7 | D3S1566 | TTCAACCCAGTCAGCTCCTTTTCGGTATTTTTCTTTTAATGAATC | ACCAGTGACGAAGGCTTGAGTCGTTGGAAATCATTCTGAGG | 59 | P3 |
| 7 | D4S1535 | TTCAACCCAGTCAGCTCCTTCACTCCCTTTTGAACTTGTGA | ACCAGTGACGAAGGCTTGAGTAGGCGAGTGTGTGAGAGCA | 59 | P3 |
| 7 | D3S1289 | TTCAACCCAGTCAGCTCCTTTGAGTTCAGCCTGGAGTCTTG | ACCAGTGACGAAGGCTTGAGTGAATGCTCCATTTTCTCCTT | 59 | P3 |
| 7 | D4S1277 | TTCAACCCAGTCAGCTCCTTGGATGTGACTGCAGACTTACGA | ACCAGTGACGAAGGCTTGAGTGCCATTCATTTGATTGAGAAC | 59 | P3 |
| 7 | D3S1267 | TTCAACCCAGTCAGCTCCTTGAGCCCAAACTTGGAGCTACT | ACCAGTGACGAAGGCTTGAGGTCACCCATACCAAGGAAGG | TD | P3 |
| 8 | D5S407 | ATCAGGGACAGCTTCAAGGATAGAGAATTTGCCCCGTCTG | GGAGTGGTGAATCCGTTAGCTTTGTTGTTCATTGGAAGTCAAG | TD | P4 |
| 8 | D6S1610 | ATCAGGGACAGCTTCAAGGAGCATTGGTGGATTGAACATTT | GGAGTGGTGAATCCGTTAGCACAGGGAGAGCAACAACCTCT | TD | P4 |
| 8 | D6S289 | ATCAGGGACAGCTTCAAGGATCTGTCCATACACCTTTCAAACA | GGAGTGGTGAATCCGTTAGCAGGTTACAGTTCGCTGAGATCAT | TD | P4 |
| 8 | D6S1581 | ATCAGGGACAGCTTCAAGGAAGGCTCATCCATGTTTCTG | GGAGTGGTGAATCCGTTAGCTGCATTCCCACATTTACTG | TD | P4 |
| 8 | D6S422 | ATCAGGGACAGCTTCAAGGAAACCCAGTCCCTGAGTGATTC | GGAGTGGTGAATCCGTTAGCGTGTGTCCAGCTGAGAGTTCC | TD | P4 |
| 8 | D5S419 | ATCAGGGACAGCTTCAAGGAGCCTCTCCAACACTTTGACTG | GGAGTGGTGAATCCGTTAGCTAGTTGCCCCTCATCACCTC | TD | P4 |
| 8 | D5S424 | GGCATTGACCCTGAGTGATTGGGTACATGGGAGTTCATTAGG | ACTGATGCCTCCGTGTAAGGTCTCATGCTGGCAGGGATA | TD | P5 |
| 8 | D5S644 | GGCATTGACCCTGAGTGATTGCGCTATTATCAGAGCCCTTT | ACTGATGCCTCCGTGTAAGGAAGGAAGCCCTGCAAGTTATC | TD | P5 |
| 8 | D6S281 | GGCATTGACCCTGAGTGATTGCCCTCCTTAGCTCCAGAAT | ACTGATGCCTCCGTGTAAGGCGTGAAACTAACTAGGGTGCCTAT | TD | P5 |
| 8 | D5S422 | ACGTAGCGATAGCGGAGTGTGAGCAAGGTCCTGTCTGAAAA | GAGTCAGTGAGCGAGGAAGCCATTAATTGATCTGGGCTGGA | TD | P6 |
| 8 | D5S406 | ACGTAGCGATAGCGGAGTGTCCTGCCAATACTTCAAGAAA | GAGTCAGTGAGCGAGGAAGCGGGATGCTAACTGCTGACTA | TD | P6 |
| 8 | D5S400 | ACGTAGCGATAGCGGAGTGTGCCTGGCTGATAGAATGAGA | GAGTCAGTGAGCGAGGAAGCTTCCTAATTTGCTGGCTTCC | TD | P6 |
| 8 | D6S309 | ACGTAGCGATAGCGGAGTGTAATTTCATCCTTGTGCGAATG | GAGTCAGTGAGCGAGGAAGCAAACCTGAAAGGCAGAGGTTG | TD | P6 |
| 8 | D5S433 | ACGTAGCGATAGCGGAGTGTTTCATTTCATCAAAATCAACAGC | GAGTCAGTGAGCGAGGAAGCCCCCATCTTTCCCATTTCTAA | TD | P6 |
| 8 | D6S262 | ACGTAGCGATAGCGGAGTGTGGAGAATCAGCCTCCTGATAAA | GAGTCAGTGAGCGAGGAAGCCCAGAATCAAGTAATGGCAAGA | TD | P6 |
| 9 | D6S264 | GACAGCATCGCCAGTCACTAAGCTGACTTTATGCTGTTCCTC | GCGTAGAGGATCCACAGGACTCAGGTCTGCAGTAGCAGAAG | TD | P1 |
| 9 | D6S1617 | GACAGCATCGCCAGTCACTAGCTACTGCAAAACAGGCACA | GCGTAGAGGATCCACAGGACCCCCCTCATTGGCATATAAA | TD | P1 |
| 9 | D6S1660 | GACAGCATCGCCAGTCACTAATCCTTTTGCCTCTCTGTGC | GCGTAGAGGATCCACAGGACCACCTTGGAGAAAGGAAACG | TD | P1 |
| 9 | D6S308 | GACAGCATCGCCAGTCACTAACAAAGGCCTTGGAGAGAAT | GCGTAGAGGATCCACAGGACGGCCTCTCTGGGTCTTCTTT | TD | P1 |
| 9 | D5S408 | GACAGCATCGCCAGTCACTACACAACTTCCAACCCTGAGA | GCGTAGAGGATCCACAGGACCAAAGCTGGTCTCGAACTCC | TD | P1 |
| 9 | D6S292 | GCTTCCTAATGCAGGAGTCGATGTCCTCTCCTTCCCACCT | GTGCAAGATTCCGAATACCGAAGAACTAAAGTTGCCTGTTCTTG | TD | P2 |
| 9 | D6S287 | GCTTCCTAATGCAGGAGTCGTCTATAGCCTACAAGGATTTCCAA | GTGCAAGATTCCGAATACCGCGGTTCCAATAGTTCCAAAC | TD | P2 |
| 9 | D5S426 | GCTTCCTAATGCAGGAGTCGTTTCTGAAAAGTGGGCAACA | GTGCAAGATTCCGAATACCGGGGTTCCCTACATTTGGATG | TD | P2 |
| 9 | D6S434 | GCTTCCTAATGCAGGAGTCGCAGGTAGTCCCCCAAAATCA | GTGCAAGATTCCGAATACCGTCGGATCATAAAACTGCCTTT | TD | P2 |
| 9 | D7S657 | GCTTCCTAATGCAGGAGTCGCAGTTTTTGGCATCCTTCTCA | GTGCAAGATTCCGAATACCGTGGTTTGCATGACTAGAAAGGTT | TD | P2 |
| 9 | D7S2465 | TTCAACCCAGTCAGCTCCTTTGAGACTCCATCTCAAAGAAAAA | ACCAGTGACGAAGGCTTGAGTGTATTAGAAATCAAAACAATTTGGAG | TD | P3 |
| 9 | D5S641 | TTCAACCCAGTCAGCTCCTTAGGTCAAGCTGTCAGGGAAA | ACCAGTGACGAAGGCTTGAGGGCATTGTTTGAGAAGTCCA | TD | P3 |
| 9 | D6S257 | TTCAACCCAGTCAGCTCCTTGAACTTTGCTAGGTGCTTGATTT | ACCAGTGACGAAGGCTTGAGAATGAGAAAATGTTCAGGCTAAAG | 59 | P3 |
| 9 | D5S1981 | TTCAACCCAGTCAGCTCCTTACACCCCTGTACCAATCCAT | ACCAGTGACGAAGGCTTGAGCGTCTCTGTGCTGACCTCCT | TD | P3 |
| 9 | D6S1697 | TTCAACCCAGTCAGCTCCTTTGCTTTAGCCCAGGAGTTTG | ACCAGTGACGAAGGCTTGAGAGGAAGTTGCTGAGCAGGAG | 59 | P3 |
| 10 | D5S2027 | ATCAGGGACAGCTTCAAGGAAGTCTAGAGATTTGCTGTTATTTTATCA | GGAGTGGTGAATCCGTTAGCAAATTTTACCACCTCATTGACTG | TD | P4 |
| 10 | D7S516 | ATCAGGGACAGCTTCAAGGACCTAGGGAGAAAGGAGACCAC | GGAGTGGTGAATCCGTTAGCCTATGGACCATGGGTTTTGG | TD | P4 |
| 10 | D5S436 | ATCAGGGACAGCTTCAAGGATGAGGGGAGAAAGAAGAAAGG | GGAGTGGTGAATCCGTTAGCAACCCCTTTCCCTGAAAAATC | TD | P4 |
| 10 | D6S460 | ATCAGGGACAGCTTCAAGGAGCCAATTCCCATTTGAAGAA | GGAGTGGTGAATCCGTTAGCTTTTCCTTCTGCATGTTCCAC | TD | P4 |
| 10 | D5S410 | ATCAGGGACAGCTTCAAGGATCAACTGGTTGATAGAACTTGAGAA | GGAGTGGTGAATCCGTTAGCGAGAAAATGTCATGCCACACA | TD | P4 |
| 10 | D7S630 | ATCAGGGACAGCTTCAAGGATGTTTCCAAAGATCAAACAGCTT | GGAGTGGTGAATCCGTTAGCTGCCTCAACTGAGTCTCATCA | TD | P4 |
| 10 | D5S418 | GGCATTGACCCTGAGTGATTACCCCAATAACCAGAAGTCCA | ACTGATGCCTCCGTGTAAGGTTGCTGAGGAAACAATGATGA | TD | P5 |
| 10 | D5S2115 | GGCATTGACCCTGAGTGATTTCCTTTCCCGTGTGATTAAGA | ACTGATGCCTCCGTGTAAGGCTTCCCTCACCCTGTTTTCC | TD | P5 |
| 10 | D5S428 | GGCATTGACCCTGAGTGATTAACATCTTAGGGCATCCTG | ACTGATGCCTCCGTGTAAGGAATGATTTAAAATAGATTAGGAGCA | TD | P5 |
| 10 | D6S462 | GGCATTGACCCTGAGTGATTGTGACATCATAGGGAGGCTG | ACTGATGCCTCCGTGTAAGGACTGGAGTTGGGGGACAT | TD | P5 |
| 10 | D5S630 | GGCATTGACCCTGAGTGATTCTGGTCTCCTGGCAATTAGG | ACTGATGCCTCCGTGTAAGGCAGAGCCACAGACAGAAGGAG | TD | P5 |
| 10 | D5S427 | ACGTAGCGATAGCGGAGTGTTCTCCTAAGGATAATTTGTTATGTTTT | GAGTCAGTGAGCGAGGAAGCTCCATAAAGTTTTGCCTCAGTG | 59 | P6 |
| 10 | D6S441 | ACGTAGCGATAGCGGAGTGTAACAATATTTGGTGACTGTTAAAGG | GAGTCAGTGAGCGAGGAAGCTGGACAAATTGATTAGGAAGTAAAG | TD | P6 |
| 10 | D5S471 | ACGTAGCGATAGCGGAGTGTTTTTCACACATTTTCCCAGC | GAGTCAGTGAGCGAGGAAGCAAAACTTCATTTACAAAAACAGGAG | TD | P6 |
| 10 | D5S416 | ACGTAGCGATAGCGGAGTGTACTCCAGCCTAGGCAATAAGA | GAGTCAGTGAGCGAGGAAGCGCTCTGAGGATCTCCTGTGTT | TD | P6 |
| 10 | D6S1721 | ACGTAGCGATAGCGGAGTGTACACGCACACACAATGGAAT | GAGTCAGTGAGCGAGGAAGCGTGTGTGCCCACCCTACTCT | 59 | P6 |
| 11 | D7S484 | CGGCATCAGAGCAGATTGTATGCGTTTGATCGAAGCAGA | AACCGTATTACCGCCTTTGAGCTGAGCAAGGCATTGTTT | TD | P7 |
| 11 | D8S264 | CGGCATCAGAGCAGATTGTAGGAACATCTGCGTCGTCTTC | AACCGTATTACCGCCTTTGAGAGAAGATGTGAATGGGAAAGC | TD | P7 |
| 11 | D8S260 | CGGCATCAGAGCAGATTGTAAGGCTTGCCAGATAAGGTTG | AACCGTATTACCGCCTTTGAGCTGAAGGCTGTTCTATGGA | 59 | P7 |
| 11 | D7S517 | CGGCATCAGAGCAGATTGTAGGTGGAGAAGCCATGTGAGT | AACCGTATTACCGCCTTTGACGTGGTTCCAATAGCAAAATC | 59 | P7 |
| 11 | 8S1784 | CGGCATCAGAGCAGATTGTACCCACCATTGAAATTAACACC | AACCGTATTACCGCCTTTGACATGATGCCTCTTACTGGTCAA | TD | P7 |
| 11 | D7S530 | GGTGATGACGGTGAAAACCTTGCATTTTAGTGGAGCACAG | CGCATATGGTGCACTCTCAGCAGGCATTGGGAACTTTG | TD | P8 |
| 11 | D8S258 | GGTGATGACGGTGAAAACCTCTGCCAGGAATCAACTGAG | CGCATATGGTGCACTCTCAGTTGACAGGGACCCACG | TD | P8 |
| 11 | D8S549 | GGTGATGACGGTGAAAACCTTGTTGTGGTTCATGGGTTG | CGCATATGGTGCACTCTCAGTTAAAACATTGCTTGGTATATATTTGA | TD | P8 |
| 11 | D7S669 | GGTGATGACGGTGAAAACCTCCTAACTAGCTGCTTAAGTATTTCTCC | CGCATATGGTGCACTCTCAGACACCATATACGGTTTACCCACA | TD | P8 |
| 11 | D7S502 | GGTGATGACGGTGAAAACCTATTGGAGATGTTGCCCTCTG | CGCATATGGTGCACTCTCAGGAGAACATACCAGCCCTGTGA | TD | P8 |
| 11 | D8S272 | GGTGATGACGGTGAAAACCTCAGGATCCCTTCATCACAAGT | CGCATATGGTGCACTCTCAGTGTTTTCTGTGCAACCTGGA | TD | P8 |
| 11 | D7S640 | GGCGCTTTCTCATAGCTCACGTCTTCCAGCCCACCC | GCCTACATACCTCGCTCTGCGCACATCACCAACAACG | TD | P9 |
| 11 | D7S513 | GGCGCTTTCTCATAGCTCACAGTGTTTTGAAGGTTGTAGGTTAAT | GCCTACATACCTCGCTCTGCATATCTTTCAGGGGAGCAGG | TD | P9 |
| 11 | D8S1771 | GGCGCTTTCTCATAGCTCACTTTACAAGAACCACCTGCC | GCCTACATACCTCGCTCTGCGATATAAAACATGACTTTGCTACCC | TD | P9 |
| 11 | D7S510 | GGCGCTTTCTCATAGCTCACGGTGTTTACCCAAAGGAGATGA | GCCTACATACCTCGCTCTGCCCCTGTTAGCCAAGATGGTC | TD | P9 |
| 11 | D8S514 | GGCGCTTTCTCATAGCTCACGGAACCTAACAGCGTCCTTTT | GCCTACATACCTCGCTCTGCGAGGCCAACAGAAAATATTCAAA | TD | P9 |
| 12 | D7S507 | CGGCATCAGAGCAGATTGTAGGCTTTCTACTAAAAACTGGTAAATGA | AACCGTATTACCGCCTTTGATCTTCAGCGAAGCTACGTACA | TD | P7 |
| 12 | D7S518 | CGGCATCAGAGCAGATTGTATGTCGTTCCAGCTACTCAGG | AACCGTATTACCGCCTTTGACTAGTAGGTGCTGGGGTGTG | 59 | P7 |
| 12 | D7S519 | CGGCATCAGAGCAGATTGTAACAGCCAAGCATTTCTGCTG | AACCGTATTACCGCCTTTGAAGCCAGAAGTGAGCCTGTTG | TD | P7 |
| 12 | D7S661 | CGGCATCAGAGCAGATTGTACAAGTGAAATGGAAACTCCTG | AACCGTATTACCGCCTTTGATGGAGCATGACCTTGGAA | TD | P7 |
| 12 | D7S486 | CGGCATCAGAGCAGATTGTATTTATATAATGTAGCATGCTGTGTGG | AACCGTATTACCGCCTTTGAGTGGCAGGTGCCTGTAATTC | TD | P7 |
| 12 | D8S285 | GGTGATGACGGTGAAAACCTGCATCACACAGAATCTTTG | CGCATATGGTGCACTCTCAGATGGGTTTATGGCCTTTAC | TD | P8 |
| 12 | D8S277 | GGTGATGACGGTGAAAACCTCCAGGTGAGTTTATCAATTCCTGAG | CGCATATGGTGCACTCTCAGTGAGAGGTCTGAGTGACATCCG | TD | P8 |
| 12 | D7S798 | GGTGATGACGGTGAAAACCTAGCTGCAAAATAGTGGAAGTAG | CGCATATGGTGCACTCTCAGCATCAATTCACATAATGACCG | TD | P8 |
| 12 | D8S284 | GGTGATGACGGTGAAAACCTGGGCATGTTACTGCATGTC | CGCATATGGTGCACTCTCAGTTTGAACACAGGTCTGCCA | TD | P8 |
| 12 | D8S505 | GGTGATGACGGTGAAAACCTCCTGCTATTTGTAGATAATGTTTGAT | CGCATATGGTGCACTCTCAGAAAACTCAAGGAAAAAGTAACTTGAC | TD | P8 |
| 12 | D7S684 | GGTGATGACGGTGAAAACCTGGTGGCAGGTGCCTATAATC | CGCATATGGTGCACTCTCAGAGATCTCTCTCTGGCCAGGTT | TD | P8 |
| 12 | D7S636 | GGCGCTTTCTCATAGCTCACAGAATTGGAGTGACTGGGCA | GCCTACATACCTCGCTCTGCTTACCCGCATCCTAGAAGCC | TD | P9 |
| 12 | D8S270 | GGCGCTTTCTCATAGCTCACACTGGGCAGGAACCTCTG | GCCTACATACCTCGCTCTGCTCCAGGGGTCTCTTAGCATC | 59 | P9 |
| 12 | D7S493 | GGCGCTTTCTCATAGCTCACGGAAGTTCCCAGCCATAGTT | GCCTACATACCTCGCTCTGCGAAAGCACTTACCTACTGAGGATTT | TD | P9 |
| 12 | D8S550 | GGCGCTTTCTCATAGCTCACCAGGAGTCAATAACCCAAAGTCAT | GCCTACATACCTCGCTCTGCTGGCACATCCCGAAGTC | TD | P9 |
| 12 | D7S531 | GGCGCTTTCTCATAGCTCACCCAACCAGAAGAGTGCCTTT | GCCTACATACCTCGCTCTGCTGGAAGACACCAGCTTTAGGA | TD | P9 |
| 13 | D11S937 | GACAGCATCGCCAGTCACTACCTCCAGACTTATTATGTGAGGAAA | GCGTAGAGGATCCACAGGACGGAACCCCTGGTGTTTCAGT | TD | P1 |
| 13 | D9S285 | GACAGCATCGCCAGTCACTATTGAAATTTGCCAAGAGAGTAGA | GCGTAGAGGATCCACAGGACCTGAAATGACCGCAATCAAG | TD | P1 |
| 13 | D11S935 | GACAGCATCGCCAGTCACTAAAAAAGTCTGAGTGAAAACCAAGTT | GCGTAGAGGATCCACAGGACTCATTCAGAAAATGTTGGCATA | TD | P1 |
| 13 | D11S987 | GACAGCATCGCCAGTCACTAGGAGGCTGAGGAAGGAGAAT | GCGTAGAGGATCCACAGGACTAGGACTCCATTTGGGGATG | TD | P1 |
| 13 | D9S1677 | GACAGCATCGCCAGTCACTATTCTCCCAGGCTTTCATTTG | GCGTAGAGGATCCACAGGACCCAGCTACTCAGGAGGCAGA | TD | P1 |
| 13 | D11S902 | GCTTCCTAATGCAGGAGTCGCCCGGCTGTGAATATACTTAATGC | GTGCAAGATTCCGAATACCGCCCAACAGCAATGGGAAGTT | TD | P2 |
| 13 | D11S904 | GCTTCCTAATGCAGGAGTCGATGACAAGCAATCCTTGAGC | GTGCAAGATTCCGAATACCGCTGTGTTATATCCCTAAAGTGGTGA | TD | P2 |
| 13 | D11S1314 | GCTTCCTAATGCAGGAGTCGCACGCACACAGACAGATCAA | GTGCAAGATTCCGAATACCGCTCCTCTGTGGTCTGGGAAA | TD | P2 |
| 13 | D11S905 | GCTTCCTAATGCAGGAGTCGAGACATCCTTGCCCATGATT | GTGCAAGATTCCGAATACCGACAGGGGCCAAATAGGTTTC | TD | P2 |
| 13 | D10S547 | GCTTCCTAATGCAGGAGTCGTCAAGACCAACCTGGCTAAGA | GTGCAAGATTCCGAATACCGCCCAATAGTCCACAGGGAGA | 59 | P2 |
| 13 | D10S249 | TTCAACCCAGTCAGCTCCTTGGCGAAATAGAGCTAACTGGTTT | ACCAGTGACGAAGGCTTGAGGGTGCCCGCTAGTATTTCG | TD | P3 |
| 13 | D9S171 | TTCAACCCAGTCAGCTCCTTAGCTAAGTGAACCTCATCTCTGTCT | ACCAGTGACGAAGGCTTGAGACCCTAGCACTGATGGTATAGTCT | TD | P3 |
| 13 | D10S192 | TTCAACCCAGTCAGCTCCTTGGTGGGAATATAACCCTTTCTT | ACCAGTGACGAAGGCTTGAGCCAGTTTCCTGCCTTTATCTC | TD | P3 |
| 13 | D9S273 | TTCAACCCAGTCAGCTCCTTGACTTCCCTCTCCTGGGCTA | ACCAGTGACGAAGGCTTGAGTGGAGTTCAATAGCTGGGTGT | 59 | P3 |
| 13 | D11S4175 | TTCAACCCAGTCAGCTCCTTGATCCAAAACCAAAAACTTGC | ACCAGTGACGAAGGCTTGAGCTCCTGGCCTCAGGTGGT | TD | P3 |
| 14 | D9S161 | ATCAGGGACAGCTTCAAGGATGCTGCATAACAAATTACCAC | GGAGTGGTGAATCCGTTAGCCATGCCTAGACTCCTGATCC | 59 | P4 |
| 14 | D9S175 | ATCAGGGACAGCTTCAAGGATGTGCTAAATACCAGAGTTGAAAGA | GGAGTGGTGAATCCGTTAGCGCCCTCTCCCAACATTTCTAC | TD | P4 |
| 14 | D10S185 | ATCAGGGACAGCTTCAAGGACACATCCTATGCTTTCATTTGC | GGAGTGGTGAATCCGTTAGCGAAACTGTGGCTAGGGTCGTAT | 59 | P4 |
| 14 | D10S197 | ATCAGGGACAGCTTCAAGGAGTAATGGCGGCACCTGTAAT | GGAGTGGTGAATCCGTTAGCGTCCTCAGGTCTCCCTCCTT | TD | P4 |
| 14 | D11S901 | ATCAGGGACAGCTTCAAGGAGCTGTGCTATGGCAAGTTTTT | GGAGTGGTGAATCCGTTAGCTTGGCAGATGTGTCTGTGAGT | TD | P4 |
| 14 | D9S287 | GGCATTGACCCTGAGTGATTAGGATGCTCCTCACGC | ACTGATGCCTCCGTGTAAGGACCACTACATTGTTCAAGGG | TD | P5 |
| 14 | D10S1653 | GGCATTGACCCTGAGTGATTCCTTTGGATAAAGCCTCCT | ACTGATGCCTCCGTGTAAGGTATCATTGTCTCATCCGGG | TD | P5 |
| 14 | D10S212 | GGCATTGACCCTGAGTGATTAAGGCATATTCAAGTGGTTGC | ACTGATGCCTCCGTGTAAGGGTTGCTGCAAAAGACGATTTC | TD | P5 |
| 14 | D10S1686 | GGCATTGACCCTGAGTGATTCTTGGCATAACCACCAGACTT | ACTGATGCCTCCGTGTAAGGTGTGGTAGAGGCAGACAAAGG | TD | P5 |
| 14 | D9S288 | ACGTAGCGATAGCGGAGTGTACCTCAACAGGGAAAAGAGGA | GAGTCAGTGAGCGAGGAAGCTCAGCTAACCTCAAATCATCCA | 59 | P6 |
| 14 | D9S157 | ACGTAGCGATAGCGGAGTGTCAGTGCCAAGCTTAAAAATGC | GAGTCAGTGAGCGAGGAAGCGTCTGAAGTCAGCAAGGCAAG | TD | P6 |
| 14 | D10S208 | ACGTAGCGATAGCGGAGTGTGTGGCATAAGTTAGCGTTCCA | GAGTCAGTGAGCGAGGAAGCGGGAGGGGCAATATTTTCAA | TD | P6 |
| 14 | D11S4046 | ACGTAGCGATAGCGGAGTGTCACTGCACTTCCACACTCCA | GAGTCAGTGAGCGAGGAAGCGCCATTGTGCATTACACCAG | 59 | P6 |
| 14 | D9S167 | ACGTAGCGATAGCGGAGTGTCCTATGTGAGTGAGAACAAGCAA | GAGTCAGTGAGCGAGGAAGCGAGCTACTGTATAAGATCAAACAATCC | TD | P6 |
| 15 | D11S4151 | CGGCATCAGAGCAGATTGTACTCCTAGTCCTCAGCACAGC | AACCGTATTACCGCCTTTGAGGGCACCTCCACCCTATT | 59 | P7 |
| 15 | D9S286 | CGGCATCAGAGCAGATTGTAAGCAGTGGCATGGAAGGA | AACCGTATTACCGCCTTTGATAGCCTCCTCTGTGCTGCTT | 59 | P7 |
| 15 | D9S1690 | CGGCATCAGAGCAGATTGTACTGCGAGTGCAAAGAGAGAA | AACCGTATTACCGCCTTTGATCGATCTCCTGACCTTGTGA | TD | P7 |
| 15 | D11S1320 | CGGCATCAGAGCAGATTGTATTTACTTATGGGGAGCTTTGACA | AACCGTATTACCGCCTTTGACAGACAGGAATTAAGGCACCA | 59 | P7 |
| 15 | D9S1793 | CGGCATCAGAGCAGATTGTATCTGCCGGGTTGTTTAAGTT | AACCGTATTACCGCCTTTGAGCCACAAACGCCAGGA | TD | P7 |
| 15 | D11S4191 | GGTGATGACGGTGAAAACCTCAGCAAGATGGCCAATTAGA | CGCATATGGTGCACTCTCAGGGAGCTCTTCCTTAGTTATTTTGGT | TD | P8 |
| 15 | D11S968 | GGTGATGACGGTGAAAACCTCCTCTTCCCCTAGGCTCTTG | CGCATATGGTGCACTCTCAGCAGCAGCCAGGATCACAAAG | TD | P8 |
| 15 | D10S591 | GGTGATGACGGTGAAAACCTACCTCGAAGGTCTGTTCTCC | CGCATATGGTGCACTCTCAGGGCTTTATGGATCATATTAATCCAC | TD | P8 |
| 15 | D11S1338 | GGTGATGACGGTGAAAACCTTGGTGTCAGAAATCTGATGGA | CGCATATGGTGCACTCTCAGTGTGACATTCACTGCCTCCT | TD | P8 |
| 15 | D9S1776 | GGTGATGACGGTGAAAACCTTTCAGAAAGGAAAGCAGACACA | CGCATATGGTGCACTCTCAGACAAAGCTCCATGCATTCCT | TD | P8 |
| 15 | D10S537 | GGCGCTTTCTCATAGCTCACTGACCTACTGTGCCTGGCTA | GCCTACATACCTCGCTCTGCGCCCCTAAATATTTGGATGAAA | TD | P9 |
| 15 | D10S189-RE1 | GGCGCTTTCTCATAGCTCACCGGCATGGGACATCTTAAAC | GCCTACATACCTCGCTCTGCGAAGCGATAGATCGATAACTGTG | TD | P9 |
| 15 | D10S587 | GGCGCTTTCTCATAGCTCACCTCCTCCTGGAAGCTCATTG | GCCTACATACCTCGCTCTGCAGGAATTCCCGCTAAACGAC | 59 | P9 |
| 15 | D11S4089 | GGCGCTTTCTCATAGCTCACAGTGCCAAAGAGATGCACAG | GCCTACATACCTCGCTCTGCCTGAGGTGGGGTGAGGATTA | TD | P9 |
| 16 | D11S898 | GACAGCATCGCCAGTCACTAAGCACCATTTGCTGAGACTG | GCGTAGAGGATCCACAGGACTGTATTTGTATCGATTAACCAACTT | TD | P1 |
| 16 | D10S217 | GACAGCATCGCCAGTCACTACCACGATGCAGAGGTT | GCGTAGAGGATCCACAGGACCCAGGTGGCTAGGAGG | TD | P1 |
| 16 | D9S1817 | GACAGCATCGCCAGTCACTAAGCTGTAGTGAGCCCTGAT | GCGTAGAGGATCCACAGGACCGTTAGGAGCCTTGAGACTT | TD | P1 |
| 16 | D9S158 | GACAGCATCGCCAGTCACTATCTCAAGCGACAACAATCAC | GCGTAGAGGATCCACAGGACGATTTGGCTAAAATAGGCTCA | TD | P1 |
| 16 | D10S196 | GCTTCCTAATGCAGGAGTCGTTCAAAGGTGGAGACCCTTC | GTGCAAGATTCCGAATACCGTTTTGGTCAGAATGGAGTGG | TD | P2 |
| 16 | D11S908 | GCTTCCTAATGCAGGAGTCGAGTGTAAAAACTCAGGCATG | GTGCAAGATTCCGAATACCGTGGATGGTAAACTCAACAAG | TD | P2 |
| 16 | D9S1682 | GCTTCCTAATGCAGGAGTCGGCTCATACTCTTGATTTCTAGGC | GTGCAAGATTCCGAATACCGAAGCAGGTCCTATTGTTATTCC | TD | P2 |
| 16 | D10S1693 | GCTTCCTAATGCAGGAGTCGACCCGGTGGGTTGCAGTC | GTGCAAGATTCCGAATACCGTGGCATGTGTGGTAGGGAGC | TD | P2 |
| 16 | D9S290 | GACAGCATCGCCAGTCACTAGCATGCCTGCATCCAT | GCGTAGAGGATCCACAGGACCCAGCCACAGCAAACC | TD | P2 |
| 16 | D10S597 | GCTTCCTAATGCAGGAGTCGGAATGAAGACATCCAGAGG | GTGCAAGATTCCGAATACCGGCAAGTATCAGAAACCCAA | TD | P2 |
| 16 | D10S1652 | TTCAACCCAGTCAGCTCCTTCGAGGAAAAGCCCAAAGG | ACCAGTGACGAAGGCTTGAGATAAGCGTGTCATGGGGAGT | TD | P3 |
| 16 | D9S283 | TTCAACCCAGTCAGCTCCTTGTGTTCTCCCAGTTCTTCAGG | ACCAGTGACGAAGGCTTGAGCAGAGAGTAGAAAGGAGAGAGAACAAT | 59 | P3 |
| 16 | D10S548 | GACAGCATCGCCAGTCACTACAGTAGTATTGAGGTATAGGGACAG | GCGTAGAGGATCCACAGGACTTGGTTGTGGTAATGGTTTC | TD | P3 |
| 16 | D9S1826 | TTCAACCCAGTCAGCTCCTTATTCATGGGAAGCTGCAAAA | ACCAGTGACGAAGGCTTGAGTCTCTGTGGGGATGTACCATT | TD | P3 |
| 16 | D10S1651 | TTCAACCCAGTCAGCTCCTTCAGCGTGAGGGAAGTTGATT | ACCAGTGACGAAGGCTTGAGTCTGCCTCTCACGTTCCTCT | 59 | P3 |
| 17 | D12S83 | ATCAGGGACAGCTTCAAGGATTTTTGGAAGTCTATCAATTTGA | GGAGTGGTGAATCCGTTAGCTAGCAGAGAAAGCCAATTCA | 59 | P4 |
| 17 | D13S218 | ATCAGGGACAGCTTCAAGGAGATTTGAAAATGAGCAGTCC | GGAGTGGTGAATCCGTTAGCGTCGGGCACTACGTTTATCT | TD | P4 |
| 17 | D12S1659 | ATCAGGGACAGCTTCAAGGAAAGTGCTGGGAACTAAACACTG | GGAGTGGTGAATCCGTTAGCCCTTCCAGAATTAGGAAAGCAA | TD | P4 |
| 17 | D13S217 | ATCAGGGACAGCTTCAAGGAGTCATCCACTTGCCTCAGC | GGAGTGGTGAATCCGTTAGCTCACGGACTCTATGATAACCTTTC | TD | P4 |
| 17 | D12S78 | ATCAGGGACAGCTTCAAGGAACGTGAGCAAAGGAACAGATG | GGAGTGGTGAATCCGTTAGCTGAACTGCTGGCTTTAACAGAA | TD | P4 |
| 17 | D13S285 | GGCATTGACCCTGAGTGATTATATATGCACATCCATCCATG | ACTGATGCCTCCGTGTAAGGGGCCAAAGATAGATAGCAAGGTA | TD | P5 |
| 17 | D12S1723 | GGCATTGACCCTGAGTGATTATCCCGCCTCTGTAGAATG | ACTGATGCCTCCGTGTAAGGAAGGCCATGTGAGCATC | TD | P5 |
| 17 | D13S170 | GGCATTGACCCTGAGTGATTTTTTGAAAAGTTCTCTCCAGACAT | ACTGATGCCTCCGTGTAAGGGCAGAATTGTGAGTAATGAAATGG | TD | P5 |
| 17 | D13S175 | ACGTAGCGATAGCGGAGTGTTATTGGATACTTGAATCTGCTG | GAGTCAGTGAGCGAGGAAGCTGCATCACCTCACATAGGTTA | TD | P6 |
| 17 | D13S263 | ACGTAGCGATAGCGGAGTGTCCTGGCCTGTTAGTTTTTATTGTTA | GAGTCAGTGAGCGAGGAAGCCCCAGTCTTGGGTATGTTTTTA | TD | P6 |
| 17 | D12S1617 | ACGTAGCGATAGCGGAGTGTAGCCTGAGGGGCCACAT | GAGTCAGTGAGCGAGGAAGCTGGGCAACTTGGATAAGAAACA | TD | P6 |
| 17 | D12S346 | ACGTAGCGATAGCGGAGTGTCCACCTGCCTGTAACACACAT | GAGTCAGTGAGCGAGGAAGCAGGTTTTGTGTTGTGGTGAGC | TD | P6 |
| 18 | D12S85 | CGGCATCAGAGCAGATTGTAGCACCTCTCACTCCATTACA | AACCGTATTACCGCCTTTGAAAATGAAAGTCAAGGGGAAC | TD | P7 |
| 18 | D12S351 | CGGCATCAGAGCAGATTGTATTGCTCTAAACTCAATATCTCAGC | AACCGTATTACCGCCTTTGAGTGCATCTGTATGTGCGTG | TD | P7 |
| 18 | D13S1265 | CGGCATCAGAGCAGATTGTATTAAGGAGCGCTGAGCAATA | AACCGTATTACCGCCTTTGACTCCATTTCCAAGTCACATCA | TD | P7 |
| 18 | D6S1582 | CGGCATCAGAGCAGATTGTAGAGCCCTTTTCCAGTTTGCA | AACCGTATTACCGCCTTTGATAGGGTCCAGGCAAGAACAG | 59 | P7 |
| 18 | D12S368 | CGGCATCAGAGCAGATTGTAAAACATGGGACAAAGGACCA | AACCGTATTACCGCCTTTGAGCAGCTCATTGCATTCATTC | TD | P7 |
| 18 | D12S86 | GGTGATGACGGTGAAAACCTAGCTAGTCTGGCATGAGCAG | CGCATATGGTGCACTCTCAGCTATCCCCTGATGATCTCCC | TD | P8 |
| 18 | D1S196 | GGTGATGACGGTGAAAACCTTGGATTCGTTACATGTTTCTCTTC | CGCATATGGTGCACTCTCAGTGGAGCTCTCATGTCTTTACATTC | TD | P8 |
| 18 | D8S279 | GGTGATGACGGTGAAAACCTACCCAGTCATTCCCTCTTCC | CGCATATGGTGCACTCTCAGTCAGGGACAAAGTGACTCGC | TD | P8 |
| 18 | D13S156 | GGTGATGACGGTGAAAACCTATTAGCCCAGGTATGGTGAC | CGCATATGGTGCACTCTCAGGCTGTGGTATGAGTTACTTAAACAC | 59 | P8 |
| 18 | D2S2368 | GGCGCTTTCTCATAGCTCACATTTGAATCTTAAAGCATCTTCCAA | GCCTACATACCTCGCTCTGCGAAAATGAGAAGCTTCGACCA | TD | P9 |
| 18 | D12S79 | GGCGCTTTCTCATAGCTCACTTGGACTGAACTGAGATGCC | GCCTACATACCTCGCTCTGCTATGTGCACCCAGACTACCA | TD | P9 |
| 18 | D12S99 | GGCGCTTTCTCATAGCTCACGCATGCAAGTCAGGGCTTAG | GCCTACATACCTCGCTCTGCCAGGGAATAGAACTTTGTCTCTCA | TD | P9 |
| 18 | D12S345 | GGCGCTTTCTCATAGCTCACGCCTGGGTAACAAAGCAAGA | GCCTACATACCTCGCTCTGCCCAGCTACTTGGGAACTCCA | TD | P9 |
| 18 | D12S336 | GGTGATGACGGTGAAAACCTTACCCTCCTCTTAGCCATGC | CGCATATGGTGCACTCTCAGTATCTTTTGGGAGCCTGCCT | TD | P9 |
| 19 | D13S158 | GACAGCATCGCCAGTCACTAGTACCCACGGAGTGAAAGAA | GCGTAGAGGATCCACAGGACGCTTTGACAATTTAGCAGCA | 59 | P1 |
| 19 | D13S173 | GACAGCATCGCCAGTCACTAAATGAAGTGGTAGCCTCATGC | GCGTAGAGGATCCACAGGACTTTGGTGCCCATAGTTTTCA | TD | P1 |
| 19 | D12S364 | GACAGCATCGCCAGTCACTACCAGTCCTGAACAGGC | GCGTAGAGGATCCACAGGACCCCTGGAAGTCCCATC | 59 | P1 |
| 7 | 3S1279 | GACAGCATCGCCAGTCACTAAGCTCATTTCCTGGTTTTGC | GCGTAGAGGATCCACAGGACTGCTGGTACATGTAGCTTACAAA | 59 | P1 |
| 19 | D13S1298 | GACAGCATCGCCAGTCACTACCTTTGACTTTCCAGAGACAGAC | GCGTAGAGGATCCACAGGACCAGTGGGACTTTCTCTGACCT | TD | P1 |
| 19 | D13S265 | GCTTCCTAATGCAGGAGTCGTCTGCCAATTACATTGCATA | GTGCAAGATTCCGAATACCGGGAGGACCCACCAACA | TD | P2 |
| 19 | D12S352 | GCTTCCTAATGCAGGAGTCGTGGACCAGGGTGATGG | GTGCAAGATTCCGAATACCGAGCAGGAGTCTGACGTTTTG | 59 | P2 |
| 19 | D12S326 | GCTTCCTAATGCAGGAGTCGAAATCACGGCACTCCATTTT | GTGCAAGATTCCGAATACCGAGAATGACCAGACCCACAGG | TD | P2 |
| 19 | D12S310 | GCTTCCTAATGCAGGAGTCGAGTGTGTTTTGCCAACAAGC | GTGCAAGATTCCGAATACCGATTCTCCAAATGCCAAAACG | TD | P2 |
| 19 | D12S324 | TTCAACCCAGTCAGCTCCTTTGACTTGGGAATTCTACCATGA | ACCAGTGACGAAGGCTTGAGGCTTGAGAAAATACCAGTAAATGC | TD | P3 |
| 19 | D13S171 | TTCAACCCAGTCAGCTCCTTGCAGATACAGACATTTTGGAAGAA | ACCAGTGACGAAGGCTTGAGGCTTGCTCTGAGCTTCTGCT | TD | P3 |
| 19 | D13S153 | TTCAACCCAGTCAGCTCCTTAGCATTGTTTCATGTTGGTG | ACCAGTGACGAAGGCTTGAGCAGCAGTGAAGGTCTAAGCC | TD | P3 |
| 20 | D14S275 | ATCAGGGACAGCTTCAAGGACATTCCCTATTCTGGACACCA | GGAGTGGTGAATCCGTTAGCGACCCTGACCCTCTCTCCTAA | TD | P4 |
| 20 | D14S280 | ATCAGGGACAGCTTCAAGGACCACACAATTAAATGCCACTACA | GGAGTGGTGAATCCGTTAGCAAGAAAAACTGTCAAACGATTTCC | TD | P4 |
| 20 | D14S292 | ATCAGGGACAGCTTCAAGGATCTCAGCATTCCCATCTTCAC | GGAGTGGTGAATCCGTTAGCTTTGAGAAACCCCTTAGCACA | TD | P4 |
| 20 | D14S258 | ATCAGGGACAGCTTCAAGGAGGTGGCTGGGGAAATAATAAA | GGAGTGGTGAATCCGTTAGCAACCAATGATGAAGTGGGTGA | TD | P4 |
| 20 | D14S985 | GGCATTGACCCTGAGTGATTCAGTGTGACCTTAAACAAGTCG | ACTGATGCCTCCGTGTAAGGCCTGTGGGGTAGATACACGA | 59 | P5 |
| 20 | D14S63 | GGCATTGACCCTGAGTGATTTGTGGAGCTAATGGTCCAGTC | ACTGATGCCTCCGTGTAAGGTCACTGGATAAGGTTGAGACTCC | 59 | P5 |
| 20 | D14S70 | GGCATTGACCCTGAGTGATTATCAATTTGCTAGTTTGGCA | ACTGATGCCTCCGTGTAAGGAGCTAATGACTTAGACACGTTGTAG | TD | P5 |
| 20 | D14S283 | GGCATTGACCCTGAGTGATTTTTCCTACCATTGATCTTCTTGC | ACTGATGCCTCCGTGTAAGGGACTATATCTCCCAGGCCTTCC | TD | P5 |
| 20 | D14S74 | GGCATTGACCCTGAGTGATTCCTGTACCACTACCTGAGTTGAGT | ACTGATGCCTCCGTGTAAGGCTTTGGCTGCCCGAAA | TD | P5 |
| 20 | D14S276 | GGCATTGACCCTGAGTGATTTGCAAGGTAGGTTGTTTTGTTT | ACTGATGCCTCCGTGTAAGGGCTCTCAGGCATTCATCAGTT | TD | P5 |
| 20 | D14S65 | ACGTAGCGATAGCGGAGTGTTCAAATCAGGACCATTTGTGG | GAGTCAGTGAGCGAGGAAGCATAAGCTCCACCCCCTAAAGA | TD | P6 |
| 20 | D14S68 | ACGTAGCGATAGCGGAGTGTGAGAGGTGGTTTTCAGTGGT | GAGTCAGTGAGCGAGGAAGCTCAGGGATAGTTGGTGGGTA | 59 | P6 |
| 20 | D14S261 | ACGTAGCGATAGCGGAGTGTACCAACTCCTTCCCACAATTT | GAGTCAGTGAGCGAGGAAGCTCTCAGGTGCCTACTTGATGC | TD | P6 |
| 20 | D14S288 | ACGTAGCGATAGCGGAGTGTAAAATTAACTTGGCATGGTGGT | GAGTCAGTGAGCGAGGAAGCCAAACAGAAAGAAATGCAGTTGA | 59 | P6 |
| 21 | D16S3068 | CGGCATCAGAGCAGATTGTATCCCTGTAGCAAAATTTCACC | AACCGTATTACCGCCTTTGATACCCCTTACTACCGCCACA | 59 | P7 |
| 21 | D16S3136 | CGGCATCAGAGCAGATTGTATTCTAGCCCTTCCCCCTTAC | AACCGTATTACCGCCTTTGATGCCATTATTGCTTTCTGGTC | TD | P7 |
| 21 | D16S515 | CGGCATCAGAGCAGATTGTATCCACCACTGCAAACCTGTA | AACCGTATTACCGCCTTTGATCCCCCAGGAGTAATTTGTTC | TD | P7 |
| 21 | D15S130 | CGGCATCAGAGCAGATTGTAAGGTGGTGCTGGCATTAGAG | AACCGTATTACCGCCTTTGACCAAAGGCAATAAGACAGGAG | TD | P7 |
| 21 | D15S1002 | GGTGATGACGGTGAAAACCTTACCCTGGTGAAGCCAGCA | CGCATATGGTGCACTCTCAGTGCTAGGTTCCCCTTTTTCCT | TD | P8 |
| 21 | D16S503 | GGTGATGACGGTGAAAACCTTTCACTTAGTTAATAAAAGGGCTACA | CGCATATGGTGCACTCTCAGGGTAGTTGTCTCCCTAATCCTGA | TD | P8 |
| 21 | D16S520-RE1 | GGTGATGACGGTGAAAACCTCAGCAACCAGCTAGCACTCA | CGCATATGGTGCACTCTCAGGGATGGAGGAATTCTGCTTG | TD | P8 |
| 21 | D15S131 | GGTGATGACGGTGAAAACCTGCTGGGCTCCTCCACTTAG | CGCATATGGTGCACTCTCAGTTTCAAGCACTCCAATCCTG | TD | P8 |
| 21 | D15S165 | GGTGATGACGGTGAAAACCTTCATTTTTGGTGGACTATAAAGGATTA | CGCATATGGTGCACTCTCAGATTCTAAAGCCCGCACGTTT | TD | P8 |
| 21 | D15S127 | GGCGCTTTCTCATAGCTCACGGGAATCCCCTCCATGAAC | GCCTACATACCTCGCTCTGCGCAGCTCAGAAATTTTCAAACA | TD | P9 |
| 21 | D16S3091 | GGCGCTTTCTCATAGCTCACACATCAACTAGCTAGCATTGACAGTAA | GCCTACATACCTCGCTCTGCTCAAATTGTTGCTAATAACACTAGGC | TD | P9 |
| 21 | D15S153 | GGCGCTTTCTCATAGCTCACCTGAAAGGGTGGGCAGAT | GCCTACATACCTCGCTCTGCGGTGGAGGCAGTGAGTACAAA | TD | P9 |
| 21 | D16S3075 | GGCGCTTTCTCATAGCTCACGCCTACCCATTCTTCACTGC | GCCTACATACCTCGCTCTGCTCCTCCACAAGTACCGAAGG | TD | P9 |
| 21 | D15S117 | GGCGCTTTCTCATAGCTCACCATAAAAGTGTGCACCAACAA | GCCTACATACCTCGCTCTGCCCTGTCGTTCAAATAAAACCTT | TD | P9 |
| 22 | D15S205 | GACAGCATCGCCAGTCACTACTTAATGGTTTGGCAGGATA | GCGTAGAGGATCCACAGGACAGCTTAAAAACAAAATCTCCC | TD | P1 |
| 22 | D16S415 | GCTTCCTAATGCAGGAGTCGCAGTCCCTGCCAACTCTCTC | GTGCAAGATTCCGAATACCGGCTTAAAATCATGGCAGAAGG | 59 | P1 |
| 22 | D16S3046 | GACAGCATCGCCAGTCACTAACATGACCTGCCAAAATTGC | GCGTAGAGGATCCACAGGACTCATGGACCCCCTATTGTTG | TD | P1 |
| 22 | D16S3103 | GACAGCATCGCCAGTCACTAGGTAGCTGGGGGTAGAGGAC | GCGTAGAGGATCCACAGGACGCCAGCAGGTCTTCCTACAG | TD | P1 |
| 22 | D16S404 | GCTTCCTAATGCAGGAGTCGCAGTCCCTGCCAACTCTCTC | GTGCAAGATTCCGAATACCGGCTTAAAATCATGGCAGAAGG | TD | P2 |
| 22 | D16S423 | GCTTCCTAATGCAGGAGTCGGCCTCCTTCACCAGATGTGT | GTGCAAGATTCCGAATACCGAGAGAGAATGTGCCTGTTTGC | TD | P2 |
| 22 | D15S1012 | GCTTCCTAATGCAGGAGTCGTGAGGCAGGACAATCACTTG | GTGCAAGATTCCGAATACCGCACTGCAGCCTTAAGTTCCTG | TD | P2 |
| 22 | D15S978 | GCTTCCTAATGCAGGAGTCGCTGGCCCCTTCTACTCACAC | GTGCAAGATTCCGAATACCGAACCTGGGACCTCTGTGTTG | TD | P2 |
| 22 | D15S120 | TTCAACCCAGTCAGCTCCTTTTTTGTGATGGTCTTTTATAGGC | ACCAGTGACGAAGGCTTGAGTCCCATCAGGAGAGTATTTTGA | TD | P3 |
| 22 | D16S516 | TTCAACCCAGTCAGCTCCTTCTGAGCATGGCTTTAGACACC | ACCAGTGACGAAGGCTTGAGCTGGAAGGCCCATAAAAAGG | 59 | P3 |
| 22 | D15S1007 | TTCAACCCAGTCAGCTCCTTCCCTCCTCATCCCATTTTTC | ACCAGTGACGAAGGCTTGAGCACGTCGATTTTCTTCTGTCTG | TD | P3 |
| 22 | D15S128 | TTCAACCCAGTCAGCTCCTTTGCATTTGTATGCAGCTGTG | ACCAGTGACGAAGGCTTGAGCTCTCTGTTTTCCTTGCCTGA | 59 | P3 |
| 22 | D15S994 | TTCAACCCAGTCAGCTCCTTCAGTGGCCCCTACATACTCC | ACCAGTGACGAAGGCTTGAGCCACCCCTTCCTACTTGTGA | TD | P3 |
| 23 | D17S949 | ATCAGGGACAGCTTCAAGGAGGAAATTGAATTGTACTTTGGTGA | GGAGTGGTGAATCCGTTAGCAGCTCTTTTTCCCTCCCTTTC | TD | P4 |
| 23 | D18S70 | ACGTAGCGATAGCGGAGTGTATTCACCATGCACACAGCAA | GAGTCAGTGAGCGAGGAAGCTGCTTGCCTTCATTCTGTTTT | TD | P4 |
| 23 | D18S478 | ATCAGGGACAGCTTCAAGGATTTCCATTAGCACTGTGGTCAG | GGAGTGGTGAATCCGTTAGCAAACAGAGAACTCTTGACAAAAAGG | TD | P4 |
| 23 | D17S1852 | ATCAGGGACAGCTTCAAGGATTCAGGAAGCTCTTCTTTGAAAT | GGAGTGGTGAATCCGTTAGCGATGGGACTCAGAAGATCACG | TD | P4 |
| 23 | D17S790 | ATCAGGGACAGCTTCAAGGACTGCTGTCGTCTGTTTGGAG | GGAGTGGTGAATCCGTTAGCTGAAGATGATGAAGGCGAAA | TD | P4 |
| 23 | D17S798 | GGCATTGACCCTGAGTGATTTCCCCATCCTTCAGAGTATAACA | ACTGATGCCTCCGTGTAAGGATCACGGTTCCATGAGAAAGA | TD | P5 |
| 23 | D17S799 | GGCATTGACCCTGAGTGATTATTGCCAGCCGTCAGTT | ACTGATGCCTCCGTGTAAGGGACCAGCATATCATTATAGACAAGC | TD | P5 |
| 23 | D17S1857 | GGCATTGACCCTGAGTGATTTGACTGCAGATCTTGGGAAGT | ACTGATGCCTCCGTGTAAGGCCCTTTTCTGCCTAAACTGCT | TD | P5 |
| 23 | D17S831 | GGCATTGACCCTGAGTGATTTGTACACCTTCCTCCTCTGGAT | ACTGATGCCTCCGTGTAAGGCCACTTTGTGAGTTTCTGAGCA | TD | P5 |
| 23 | D17S1868 | ACGTAGCGATAGCGGAGTGTTTCATGTTTGGGTGTTTTTCA | GAGTCAGTGAGCGAGGAAGCTCACAGATAAAGGGCATTTGG | 59 | P5 |
| 23 | D18S1102 | ACGTAGCGATAGCGGAGTGTTTTCAGGATTTTGGAGCC | GAGTCAGTGAGCGAGGAAGCGGAATGACTGCGTCTGTG | TD | P6 |
| 23 | D17S849 | ACGTAGCGATAGCGGAGTGTCAATTCTGTTCTAAGATTATTTTGG | GAGTCAGTGAGCGAGGAAGCCTCTGGCTGAGGAGGC | TD | P6 |
| 23 | D18S61 | ACGTAGCGATAGCGGAGTGTTCCTTCACATCTAGCACAGTGG | GAGTCAGTGAGCGAGGAAGCCCAAACCAACATAATATAGCAATGG | TD | P6 |
| 23 | D18S461 | ACGTAGCGATAGCGGAGTGTCATGCTCCCACAACTCCATA | GAGTCAGTGAGCGAGGAAGCTTTTTGCCACACCCTTTACA | 59 | P6 |
| 24 | D18S474 | CGGCATCAGAGCAGATTGTAGCACTATGGGGTGTTTACCAG | AACCGTATTACCGCCTTTGAAATGAGGGTTTGGCTTTCAA | TD | P7 |
| 24 | D18S53 | CGGCATCAGAGCAGATTGTAGGGTAGGAGGCTGTCCTGT | AACCGTATTACCGCCTTTGAAGAACTTGCTTAAGCCTTGGA | TD | P7 |
| 24 | D17S938 | CGGCATCAGAGCAGATTGTACGCCCGACCAATAAAAGCAT | AACCGTATTACCGCCTTTGAACTCATCTTGCCCTGGAACA | 59 | P7 |
| 24 | D18S464 | CGGCATCAGAGCAGATTGTATGCTACTTGGCTCCAGAAAA | AACCGTATTACCGCCTTTGAGTTGCCCTAAAAGCCACTTG | TD | P7 |
| 24 | D18S1161 | CGGCATCAGAGCAGATTGTACAGGAAGCTCCTGTGGAATC | AACCGTATTACCGCCTTTGACCAGAGTGAGGCAGGAAGTT | TD | P7 |
| 24 | D18S59 | GGTGATGACGGTGAAAACCTTCCCCAAGGAGCTTCCTATC | CGCATATGGTGCACTCTCAGCCCACTCCTGTGCACTCTCT | TD | P8 |
| 24 | D18S63 | GGTGATGACGGTGAAAACCTCCATGTTGGATGTATCAAGTCC | CGCATATGGTGCACTCTCAGTCTCCAGGAACATTGTTTTACTTT | TD | P8 |
| 24 | D17S921 | GGTGATGACGGTGAAAACCTTGGCATTTGTACATCAAAGCA | CGCATATGGTGCACTCTCAGTTGGCCTCAGACTTGGACTT | 59 | P8 |
| 24 | D17S784 | GGTGATGACGGTGAAAACCTTGGGATGAGAGGGATGAGTC | CGCATATGGTGCACTCTCAGTGTGTCTTGGTGTCCTCTGC | TD | P8 |
| 24 | D18S64 | GGTGATGACGGTGAAAACCTGGACGGGGAGCTTATTGTTT | CGCATATGGTGCACTCTCAGCAAAGAGGTCCCAAGTTCTCT | TD | P8 |
| 24 | D18S452 | GGCGCTTTCTCATAGCTCACGGGCATACATAGTGCAAACCT | GCCTACATACCTCGCTCTGCGAACCTGGAATGGTTCATTTG | TD | P9 |
| 24 | D17S928 | GGCGCTTTCTCATAGCTCACGCCTGGGTGTCAAGAGTGAG | GCCTACATACCTCGCTCTGCCCACTGGCTGCTTCCATATTA | TD | P9 |
| 24 | D17S785 | GGCGCTTTCTCATAGCTCACCAAAGCTCTGACCTGCCAAA | GCCTACATACCTCGCTCTGCAGTTACTGGGGATTGCACGA | 59 | P9 |
| 24 | D18S68 | GGCGCTTTCTCATAGCTCACCGGCACTTCCAACTCACATA | GCCTACATACCTCGCTCTGCCAGCCAGATTCTGCGTTTCT | TD | P9 |
| 24 | D17S944 | GGCGCTTTCTCATAGCTCACCTTCAGTGAGCCGTGACTGT | GCCTACATACCTCGCTCTGCTTAGGCTCGCCCTGAATAAA | 59 | P9 |
| 25 | D20S112 | GACAGCATCGCCAGTCACTAATGGGTGTGCCAAATCTC | GCGTAGAGGATCCACAGGACTTCTTGTAAGTCAGACAGCATCA | TD | P1 |
| 25 | D20S889 | GACAGCATCGCCAGTCACTATTTGGTGGAATCCTCTCCAG | GCGTAGAGGATCCACAGGACGCTCCCAGGATGTCACTAGC | TD | P1 |
| 25 | D20S117 | GACAGCATCGCCAGTCACTACTCCAGAACCAACCCTGCT | GCGTAGAGGATCCACAGGACTGTTCCTCAAACCCTTGTCC | TD | P1 |
| 25 | D19S220 | GACAGCATCGCCAGTCACTAAAAGGCCATGTCATTTGTCC | GCGTAGAGGATCCACAGGACAACACCTAGGAGGACGGCTA | TD | P1 |
| 25 | D20S171 | GCTTCCTAATGCAGGAGTCGTTTTATAGGTGAGGACCCTGAG | GTGCAAGATTCCGAATACCGCTGTCTCTGTCTGTCTCTCTCTCTC | TD | P2 |
| 25 | D19S210 | GCTTCCTAATGCAGGAGTCGCCCTGGTCTCACACTGGACT | GCTTCCTAATGCAGGAGTCGCCCTGGTCTCACACTGGACT | TD | P2 |
| 25 | D19S221 | GCTTCCTAATGCAGGAGTCGGGCGAGAGAGCAAGACTCTG | GTGCAAGATTCCGAATACCGTCACCCAGTCTCCAGTAGCA | TD | P2 |
| 25 | D20S100 | GCTTCCTAATGCAGGAGTCGTTGTGCCTTGGCTTAAGGTC | GTGCAAGATTCCGAATACCGTGTGAGCTGAATAGCCCTTG | TD | P2 |
| 25 | D20S115 | TTCAACCCAGTCAGCTCCTTCCGGAGTTAGTTTTATTATCTTGC | ACCAGTGACGAAGGCTTGAGCCTGTCCAGAACTGTCATATTG | TD | P3 |
| 25 | D20S196 | TTCAACCCAGTCAGCTCCTTTTGGTGACCCTGAGACAGAGTG | ACCAGTGACGAAGGCTTGAGAACGAACTACCTGTTGATTTGCTCC | TD | P3 |
| 25 | D19S420 | TTCAACCCAGTCAGCTCCTTGGCTGTCCTTCCACCTTCTA | ACCAGTGACGAAGGCTTGAGGGAAATGGGAGGACACTGAG | TD | P3 |
| 25 | D19S871 | TTCAACCCAGTCAGCTCCTTTCTCCCTAGGCTACTATTCTTGT | ACCAGTGACGAAGGCTTGAGCCATTCTCTGCCTCCCAAAG | TD | P3 |
| 26 | D21S266 | ATCAGGGACAGCTTCAAGGAGGGGACATTGAGTCATCACA | GGAGTGGTGAATCCGTTAGCAGGCAAATGAAGACCTGAAC | TD | P4 |
| 26 | D19S902 | ATCAGGGACAGCTTCAAGGAGGGTTTTCATGTTCTGTTTGC | GGAGTGGTGAATCCGTTAGCAGGAAGGAAGCAAGCTACTGG | TD | P4 |
| 26 | D20S107 | ATCAGGGACAGCTTCAAGGACTACATGATGCCTCTTGGGA | GGAGTGGTGAATCCGTTAGCTCAGACAATGGCAAATTCCT | TD | P4 |
| 26 | D20S119 | ATCAGGGACAGCTTCAAGGATGTAAGGCTTTTCCCCCTCTA | GGAGTGGTGAATCCGTTAGCTGCAGCAAAAGCTAACTGACA | TD | P4 |
| 26 | D20S186 | ATCAGGGACAGCTTCAAGGACTAAGTGGATGGGCATAGCTC | GGAGTGGTGAATCCGTTAGCTTGTTCACAGGATCGCTAAAT | TD | P4 |
| 26 | D22S420 | GGCATTGACCCTGAGTGATTGTACTGGGGAGGGCGTTATC | ACTGATGCCTCCGTGTAAGGCTCATGTGGTCGCTGTGTTC | TD | P5 |
| 26 | D22S280 | GGCATTGACCCTGAGTGATTGCTCCAGCCTATCAGGATG | ACTGATGCCTCCGTGTAAGGGATTCCAGATCACAAAACTGGT | TD | P5 |
| 26 | D19S216 | GGCATTGACCCTGAGTGATTTGTCACTCTAACTCCGCTTTGA | ACTGATGCCTCCGTGTAAGGTCAAACTCCTAGCCTCAAGCA | TD | P5 |
| 26 | D22S423 | GGCATTGACCCTGAGTGATTCTCCCAGCTGAAAACAAAATG | ACTGATGCCTCCGTGTAAGGTTGAGTGGATGAAGAGTGAGTGA | TD | P5 |
| 26 | D22S539 | ACGTAGCGATAGCGGAGTGTAGGCTGTATGCATTTTTGCAC | GAGTCAGTGAGCGAGGAAGCTCATACCAATGCAATATGAAACG | TD | P6 |
| 26 | D21S1252 | ACGTAGCGATAGCGGAGTGTAATGGCTTAAAATATGCAGGATCT | GAGTCAGTGAGCGAGGAAGCTCTTACATCTCCTAGGGTGAGGAC | TD | P6 |
| 26 | D19S884 | ACGTAGCGATAGCGGAGTGTCACACATTGGCTCACACAAC | GAGTCAGTGAGCGAGGAAGCATGCCCTCAGGACCCC | TD | P6 |
| 26 | D22S274 | ACGTAGCGATAGCGGAGTGTAAATGGGAGGACTGCTTGAGT | GAGTCAGTGAGCGAGGAAGCCTTCACCAGCTTGCTAAATCG | TD | P6 |
| 27 | D20S195 | CGGCATCAGAGCAGATTGTATCTGACAATTCCAGATCCTCA | AACCGTATTACCGCCTTTGATGCAGGAAGCTTGACAAATG | TD | P7 |
| 27 | D22S315 | CGGCATCAGAGCAGATTGTATCTCCCTTCCTTCTTGCCTA | AACCGTATTACCGCCTTTGAACTTGTTGCCAGGAGAAAGC | TD | P7 |
| 27 | D19S571 | CGGCATCAGAGCAGATTGTAGGGCAGAGGTAGGAGCATC | AACCGTATTACCGCCTTTGAGGGGACTTAGCCAAACTTGA | TD | P7 |
| 27 | D19S209 | CGGCATCAGAGCAGATTGTAGGGGGATTTTCCAATCAGAG | AACCGTATTACCGCCTTTGACTCTGGGGAGTCGGAGGAG | TD | P7 |
| 27 | D19S418 | GGTGATGACGGTGAAAACCTCCAGGCATCCAGTGTTTTATC | CGCATATGGTGCACTCTCAGCCTCTGCCCAGTTTCCTTATG | TD | P8 |
| 27 | D20S173 | GGTGATGACGGTGAAAACCTGGCAAGGTCTAAGCAGGTGA | CGCATATGGTGCACTCTCAGGGCAGAACCAAAGACTCGTG | TD | P8 |
| 27 | D21S1914 | GGTGATGACGGTGAAAACCTCCCAAACCAAAATTGAGTGTTA | CGCATATGGTGCACTCTCAGGAACCAGGGCATGTAAACAAA | TD | P8 |
| 27 | D21S1908 | GGTGATGACGGTGAAAACCTCATGTGTCTTGGTCTTCTCACC | CGCATATGGTGCACTCTCAGCTATGAATCTCTGGCTGGACA | TD | P8 |
| 27 | D22S283 | GGCGCTTTCTCATAGCTCACCAGCATCATCATCTACCACTTAAAA | GCCTACATACCTCGCTCTGCGGAGCTCGGGACTTTCTG | 59 | P9 |
| 27 | D21S1256 | GGCGCTTTCTCATAGCTCACAGAAAGAAGTAAAAAGCCTATGGTC | GCCTACATACCTCGCTCTGCCCTGACATTGGAACATAAAAATC | TD | P9 |
| 27 | D20S178 | GGCGCTTTCTCATAGCTCACGCACAATGTGAACATGCGTA | GCCTACATACCTCGCTCTGCCTGGGATGCAGGGAAAAC | 59 | P9 |
| 27 | D19S226 | GGCGCTTTCTCATAGCTCACCAGCTCCTGCCTAAGTTTCC | GCCTACATACCTCGCTCTGCCAGTTGGTCCAGGATTTGAA | TD | P9 |

* Amplification Protocol: TD = 68-60 Touchdown Method (C), 59 = 59 degree Method (C)

**B. Primers with fluorescent tags**

| **Tag Primer** | **Sequence (5'>3')** |
| --- | --- |
| P1 | 6FAMGACAGCATCGCCAGTCACTA |
| P2 | VICGCTTCCTAATGCAGGAGTCG |
| P3 | NEDTTCAACCCAGTCAGCTCCTT |
| P4 | 6FAMATCAGGGACAGCTTCAAGGA |
| P5 | VICGGCATTGACCCTGAGTGATT |
| P6 | NEDACGTAGCGATAGCGGAGTGT |
| P7 | 6FAMCGGCATCAGAGCAGATTGTA |
| P8 | VICGGTGATGACGGTGAAAACCT |
| P9 | NEDGGCGCTTTCTCATAGCTCAC |

**C. Amplification Protocol**

Amplification of microsatellite markers was carried out in two steps, with markers being divided between one of two methods for each step. In the first step, unlabeled primers were used to amplify the regions surrounding each marker. Each of these primers included extensions designed to be homologous to fluorescently tagged primers used in the second step.

The first step PCR was performed using two approaches, depending on which was most successful for that primer. For the touchdown approach, 10μL reaction volumes containing 40 ng of genomic DNA, 1 μl of a mixture of 5 μM each forward (F) and reverse (R) primers as shown in A (above), 1 μl GeneAmp 10×PCR Gold buffer (Applied Biosystems), 0.6 μl of 25 mM MgCl2, 0.4 μl of 2.5 mM dNTP mixture, and 1 μl of 5U/μl Taq DNA polymerase were used. The touchdown PCR amplification consisted of a denaturing step at 94°C for 5 min, followed by 30 cycles of amplification (94°C for 30s, annealing temperature for 30s, and 72°C for 30s. The annealing temperature was initially set at 68° and decreased in a stepwise fashion by 1°C every cycle for 8 cycles, then maintained at 60°C for 22 cycles. Cycling was followed by a final extension at 72°C for 10 min. For the second step 1 μl aliquots of the first step reactions were used directly as template in the same reaction and amplification scheme except using 0.5 μl of mixed fluorescently labeled primers as listed in (B).

For the 59° approach, 5μL reaction volumes containing 40 ng of genomic DNA, 0.33 μl of a mixture of 5 μM each forward (F) and reverse (R) primers as shown in A (above), 0.5 μl GeneAmp 10×PCR Gold buffer (Applied Biosystems), 0.5 μl of 25mM MgCl2, 0.5 μl of 2.5 mM dNTP mixture, and 0.5 μl of 5U/μl Taq DNA polymerase were used. The 59° PCR amplification consisted of a denaturing step at 94°C for 5 min, followed by 30 cycles of amplification (94°C for 30s, 59° for 30s, and 72°C for 30s). Cycling was followed by a final extension at 72°C for 10 min. For the second step 0.5 μl aliquots of the first step reactions were used directly as template in the same reaction and amplification scheme except using 0.33 μl of mixed fluorescently labeled primers as listed in (B).

After the second step amplification 2 ul of mixed panel PCR products were added to 7.75 μl of Hi-Di formamide (Life technologies) and 0.25 μl of GeneScan™ 600 LIZ® dye Size Standard and were denatured at 95°C for 5 min before being analyzed on an ABI 3130 Genetic Analyzer (Applied Biosystems).
