# Supplementary material for: A novel LRAT mutation affecting splicing in a family with early onset retinitis pigmentosa
Source: Hum Genomics. 2018 Jul 4;12:35. doi: 10.1186/s40246-018-0165-3 (PMC6033202; doi:10.1186/s40246-018-0165-3)
Supplement: Supplementary file 2 — Table S2. Primer list (DOCX 12 kb). [file 40246_2018_165_MOESM2_ESM.docx]

**Table S2. Primer list.**

| Name | Sequence | Purpose |
| --- | --- | --- |
| LRAT-E2-F | GTACTTTGCGCCGTACCTCA | *LRAT* sequencing |
| LRAT-E2-R | TAGGGGAGGTGTCCAGGAAT | *LRAT* sequencing |
| LRAT-E3-F | ACGGTATAGAATCAGAACTGGAC | *LRAT* sequencing |
| LRAT-E3-R | TTGTTCTTGGGCTTACACAGT | *LRAT* sequencing |
| LRAT-gDNA-F | GTCCTCCTTTGCCTTCCTCT | PCR amplification for cloning |
| LRAT-gDNA-R | ATTCTACCACACCCTGTCGT | PCR amplification for cloning |
| LRAT-gDNA-F-SalI | CGGGTCGACGTCCTCCTTTGCCTTCCTCT | PCR amplification for cloning |
| LRAT-gDNA-R-KpnI | CGGGGTACCATTCTACCACACCCTGTCGT | PCR amplification for cloning |
| pET01-Q5SDM-F | CTCCCTGGCATTGGCACAACT | mutagenesis |
| pET01-Q5SDM-R | GTCATGTCAGCACAGGGC | mutagenesis |
| pET01-5' exon-F | GAGGGATCCGCTTCCTGCCCC | Amplification of cDNA |
| pET01-3' exon-R | CTCCCGGGCCACCTCCAGTGCC | Amplification of cDNA |
| pCMV-F | GCACGCAGAAGGTGGTCT | Amplification of cDNA |
| pCMV-R | CCGCTTACTTGTCATCGTCG | Amplification of cDNA |
